# 5-Azacytidine Potentiates Anti-tumor Immunity in a Model of Pancreatic Ductal Adenocarcinoma

**DOI:** 10.3389/fimmu.2020.00538

**Published:** 2020-03-31

**Authors:** Nancy D. Ebelt, Edith Zuniga, Benjamin L. Johnson, Don J. Diamond, Edwin R. Manuel

**Affiliations:** ^1^Department of Immuno-Oncology, Beckman Research Institute, City of Hope, Duarte, CA, United States; ^2^Department of Hematology and Hematopoietic Stem Cell Transplantation, City of Hope, Duarte, CA, United States

**Keywords:** pancreatic ductal adenocarcinoma, transposable element, 5-azacytidine, anti-tumor immunity, tumor-associated antigens, immune evasion

## Abstract

Tumors evolve a variety of mechanisms to escape immune detection while expressing tumor-promoting molecules that can be immunogenic. Here, we show that transposable elements (TE) and gene encoded, tumor-associated antigens (TAA), which can be both highly immunogenic and tumor-promoting, are significantly upregulated during the transition from pre-malignancy to malignancy in an inducible model of pancreatic ductal adenocarcinoma (PDAC). Coincident with the increased presence of TEs and TAAs was the downregulation of gene transcripts associated with antigen presentation, T cell recruitment and intrinsic anti-viral responses, suggesting a unique strategy employed by PDAC to possibly augment tumorigenesis while escaping detection by the immune system. *In vitro* treatment of mouse and human PDAC cell lines with the DNA methyltransferase inhibitor 5-azacytidine (Aza) resulted in augmented expression of transcripts for antigen presentation machinery and T cell chemokines. When immunocompetent mice implanted with PDAC were therapeutically treated with Aza, we observed significant tumor regression that was not observed in immunocompromised mice, implicating anti-tumor immunity as the principal mechanism of tumor growth control. Analysis of PDAC tumors, immediately following Aza treatment in immunocompetent mice, revealed a significantly greater infiltration of T cells and various innate immune subsets compared to control treatment, suggesting that Aza treatment enhances tumor immunogenicity. Thus, augmenting antigen presentation and T cell chemokine expression using DNA methyltransferase inhibitors could be leveraged to potentiate adaptive anti-tumor immune responses against PDAC.

## Introduction

Tumorigenesis involves extensive epigenetic reprogramming as cells transform and evolve. Most unmethylated regions, encoding tumor suppressors for instance, become heavily methylated whereas genes that are typically silenced become hypomethylated (1). Some genes that are hypomethylated during cancer progression have oncogenic or tumor-promoting activity such as certain cancer germline/cancer testis genes (2) and transposable elements (TEs) (3). The majority of TEs with activity in the setting of cancer fall within the class I retrotransposable elements, which includes long terminal repeats (LTRs), endogenous retroviruses (ERVs), long interspersed elements (LINEs), short interspersed elements (SINEs), and *Alu* repeats (4–6). In humans, LINE-1 elements (autonomous retrotransposons), as well as the non-autonomous SINE and *Alu* repeats, are active in the genome as evidenced by various incidences of disease caused by their insertions (6, 7). In human cancers, LINE-1 hypomethylation correlates with worse overall prognosis (5) and activity from HERVK (HML-2), which is typically silenced in adult tissues, has been detected (8–12).

In addition to the tumor-promoting activities of many of the molecules that would be expressed during tumor hypomethylation, they could also be immunogenic. Proteins that are relatively restricted to tumor cell expression or that are more highly expressed by tumor cells, termed tumor-associated antigens (TAAs), can encode immunogenic epitopes that are processed and presented by MHC class I molecules to induce adaptive immunity (13). A few studies have identified TE-derived proteins that may act as antigens (3, 12), and TE expression alone has been shown to initiate innate (cell-intrinsic) anti-viral immunity. Reverse transcription of transcripts from Class I retrotransposable elements in adult tissues can produce dsDNA that stimulates interferon (IFN) responses through “viral mimicry” (14, 15). Immunity to TAAs and TEs thus represents an opportunity for development of anti-cancer therapies (14–17).

Despite high expression of potentially immunogenic TAAs or TEs, tumors typically do not spontaneously regress due to concurrent development of mechanisms that allow immune escape. In various cancers, IFN-γ response genes and genes that encode major histocompatibility (MHC) class molecules and other antigen presentation machinery can be hypermethylated or mutated leading to reduced tumor immunogenicity (18–20). Therefore, methylation of immune response-related genes may be a source of selection for cells that have increased expression of TAAs and TEs during tumorigenesis. The DNA methyltransferase inhibitors (DNMTi) 5-azacytidine (Aza) and 5-aza-2′-deoxycytidine (Dac) have shown efficacy in various pre-clinical models of cancer and are currently FDA-approved for the pre-leukemic disorder myelodysplastic syndrome (MDS) (21). Mechanisms of action include reversal of abnormal DNA promoter methylation leading to re-expression of silenced genes including tumor suppressors, and changes to cancer signaling pathways including apoptosis, cell cycle activity, and stem cell functions (22–24). Recent key studies have revealed that lower-dose treatments with DNMTi induce an anti-tumor immune response through increased expression of dsDNA intermediates of transposable elements or immune response genes (14, 15). Interestingly, increased MHC I expression after DNMTi treatment, along with increased expression of anti-viral response genes, has been observed coincident with the regression of breast cancer and melanomas (25). Thus, DNMTi treatment as an anti-cancer therapy should be further studied for their potential to stimulate anti-tumor immune responses.

In this study, we identify TE families and TAAs upregulated during the transition from non-malignant acinar-ductal metaplasia (ADM) to malignant pancreatic ductal adenocarcinoma (PDAC) in a spontaneous mouse model of pancreatic cancer. In addition, transition to malignancy is associated with downregulation of genes involved in antigen presentation, T cell recruitment and anti-viral immunity. We confirm that treatment of PDAC cells, with the DNMTi 5-Azacytidine (Aza), results in the induction of gene transcripts involved in antigen presentation and T cell recruitment, which likely contributes to tumor growth control observed *in vivo*. Therapeutic Aza treatment correlated with an overall increase in tumor immune infiltrate with specific increases in CD4 and CD8 T cells, indicating an important role for adaptive anti-tumor immunity in tumor control. These data support the potential use of DNMTi as a therapeutic strategy to enhance anti-tumor immunity in PDAC and various other malignancies.

## Materials and Methods

### Animal Strains and Husbandry

Experiments involving animals were performed according to the standards of the Institution Animal Care and Use Committee (IACUC) at the City of Hope. *Ptf1a*^*tm*2(*cre*/*ESR*1)*Cvw*/^*J* (*Ptf1a*^*CreERTM*)^) mice (26) (Jackson Laboratories) were crossed to *Kras*^*LSL*−*G*12*D*/+^*; Trp53*^*R*270*H*/+^ *Rosa26*^*LSL*−*mCL*/*LSL*−*mCL*^ mice provided by Dr. Thomas Ludwig (Ohio State University) (27), to generate *Ptf1a*^*CreERTM*/+^; *Kras*^*LSL*−*G*12*D*/+^*; Trp53*^*LSL*−*R*270*H*/+^*; Rosa26*^*LSL*−*mCL*/*LSL*−*mCL*^(KPT) mice. KPT mice were maintained on a B6(Cg)-*Tyr*^*c*−2*J*^/J, (B6-albino) background. Tamoxifen (Sigma-Aldrich, Cat# T5648) was given at 4–5-week of age through intraperitoneal injections at 2 mg tamoxifen/mouse every other day for 5 days. The KPT277 tumor cell line comes from a KPT tumor that was minced and allowed to grow in culture until a cell line was established.

### Reagents and Cell Lines

PANC-1, MiaPaCa-2, and Hs-766-T cell lines were obtained from ATCC (CRL-1469, CRL-1420, and HTB-134, respectively). All cell lines were maintained in DMEM (Corning, 10-013-CV) supplemented with L-Glutamine plus Penicillin/Streptomycin (1×) (Gibco, 10378-016) and 10% FBS (Gemini Bio-products, 100–500). The mouse pancreas adenocarcinoma cell line Pan02 (also known as Panc02) was kindly provided by Dr. DC Linehan, Washington University School of Medicine. Pan02 was cultured in RPMI-1640 medium (Corning, 10-040-CV) supplemented with L-Glutamine plus Penicillin/Streptomycin (1×) (Gibco, 10378-016) and 10% FBS (Gemini Bio-products, 100–500). 5-azacytidine was purchased from EMD Millipore (5.04317.0001). 3-(4,5-dimethylthiazol-2-yl)-2,5-diphenyltetrazolium bromide (MTT) reagent was purchased from Acros (AC158990010). Anti-PD-1 antibody (J43) was purchased from Bio X Cell (BE0033-2).

### Tissue Immunofluorescence

Deparaffinized and rehydrated slides were incubated in MaxBlock Autofluorescence Reducing Reagent A (MaxVision Biosciences) for 5 min. Antigen retrieval was performed using 10 mM sodium citrate with 0.05% Tween 20, pH 6.0 heated to 95°C for 20 min. Slides were blocked in 5% donkey serum (Jackson ImmunoResearch, 017-000-001) in PBS-T. Primary antibody was incubated on slides overnight at 4°C. Secondary antibody was incubated on slides for 1 h at room temperature followed by MaxBlock™ Autofluorescence Reducing Reagent B for 5 min. Nuclei were counterstained with 4′,6-Diamidino-2-Phenylindole Dihydrochloride.

The following primary antibodies and dilutions were used: Cpa1 1/1,000 (R&D Systems Cat# AF2765, RRID:AB_2085841), Sox9 1/5,000 (Millipore Cat# AB5535, RRID:AB_2239761), and CK19 1/20 (developed by Rolf Kemler, Max Planck Institute, Freiburg, Germany was obtained from the Developmental Studies Hybridoma Bank, created by the NICHD of the NIH and maintained at The University of Iowa, Department of Biology).

### RNA-seq and Gene Expression Analysis

RNA-Seq libraries were prepared from laser capture microdissection (LCM) samples from regions of healthy pancreas, ADM, and PDAC. Ten micron sections of KPT pancreatic tissue were cut and adhered to PEN membrane slides (Leica, 11600289). Slides were fixed in 70% ethanol at −20°C and nuclei were visualized by staining with hematoxylin QS (Vector Labs, H-3404). Stained slides were immediately subjected to microdissection using a Leica LMD 7000 microscope equipped with a 349 nm solid state LASER and CC7000 digital camera. RNA samples were prepared from LCM sections using the Kapa RNA HyperPrep Kit with RiboErase (Kapa Biosystems, KR1351) followed by ribosomal RNA depletion, purification, and DNase treatment to remove the hybridization oligonucleotides. Purified RNA was fragmented followed by first-strand cDNA synthesis, second-strand cDNA synthesis, and 3′ end adenylation. Barcoded adaptors were added to double-stranded cDNA fragments. Thirteen cycles of PCR were performed to produce the final sequencing library. Library templates were prepared for sequencing using the HiSeq SR Cluster v4 Kit (Illumina, GD-401-4001). Sequencing runs were performed using the Illumina HiSeq 2500 platform with HiSeq SBS v4 Kit (Illumina, FC-401-4002). The HiSeq Control (HCS 2.2.38) and Real-Time Analysis (RTA 1.18.61) software were used for image analysis and base calling. Sequenced reads were aligned to the mouse mm10 reference genome using TopHat2 (28) and transcript expression levels were quantified by HTSeq (29) and DESeq2 (30). Transcripts were quantified as reads per kilobase of transcript per million fragments mapped (RPKM). Complete raw and processed RNA-Seq data is available online through Gene Expression Omnibus (https://www.ncbi.nlm.nih.gov/geo/, Accession Number GSE111540).

### Repeat Element Data Analysis From RNA-seq

Transposable element (TE) expression was estimated as described in Pezic et al. (31). Briefly, RNA-seq libraries were first mapped to rRNA sequences (GenBank identifiers: 18S, NR_003278.3; 28S, NR_003279.1; 5S, D14832.1; and 5.8S, K01367.1) with Bowtie 0.12.7 (32). rRNA-depleted data was then aligned to the mouse genome (mm10) with Bowtie 0.12.7 allowing 0 mismatches and up to 10,000 positions. RepeatMasker annotation tables were obtained from the University of California at Santa Cruz (UCSC) genome browser (33). Numbers of reads aligning to individual RepeatMasker-annotated genome regions were calculated accounting for multiple mapping positions. Mappability-corrected read numbers corresponding to TE and other repetitive elements were aggregated by class (repClass) and normalized as RPKM mapped reads to the genome.

### Methyl-Specific PCR

Total genomic DNA was isolated from cells *in vitro* using the QIAamp DNA Mini Kit (51304, Qiagen, Venlo, Netherlands). Five hundred nanograms of total DNA was subjected to bisulfite conversion using “Protocol A” (EpiJET Bisulfite Conversion Kit, K1461, ThermoFisher Scientific). DMSO and Aza-treated, bisulfite-converted DNA, was subjected to 45 rounds of PCR at 48°C annealing temperature using the EpiTECT MSP Kit (59305, Qiagen). The −2,000–0bp promoter sequence for murine IAP was put into MethPrimer (34) to identify CpG islands and construct primers to detect methylated or unmethylated DNA. Unmethylated IAP L: GTTTGGTTAGAGGGAGTAGAGAGTAGT; Unmethylated IAP R: ATCCTAAACCAACCTAAAAAACAAA; Methylated IAP L: TTTGGTTAGAGGGAGTAGAGAGTAGC; Methylated IAP R: TATCCTAAACCGACCTAAAAAACG. Percent unmethylated DNA was calculated by dividing band densities (quantified with ImageJ, NIH) for unmethylated DNA by the total density for unmethylated plus methylated DNA per sample.

### Quantitative PCR

Total RNA was isolated using the Omega Biotek E. Z. N. A. Total RNA kit I with on column DNase I digestion of remaining genomic DNA (R6834-01). Total RNA was converted to cDNA using the Applied Biosystems High Capacity cDNA Reverse Transcription Kit (using random primers) (4368814). cDNA was amplified and fluorescently labeled using the Bioland Scientific 2× qPCR Master Mix (Low Rox) (QP02-02) containing SYBR Green in the Applied Biosystems QuantStudio3 Real Time PCR System. Experiments were run with “no template” controls to control for nucleic acid contamination in water or primers, no data was used if sample CTs fell within five CTs of the “no template” controls. Primers used can be found in Supplementary Table 3.

### Western Blot

Protein lysates from cell lines were prepared by resuspension in RIPA lysis buffer (ThermoFisher, 89900) followed by freezing at −20°C. Protein lysates from tumors were prepared by homogenization of flash frozen tumor tumors in RIPA buffer using Dounce homogenizers. Genomic DNA was sheared by centrifuging lysate in a QIAshredder column (Qiagen #79654). Protein assay was performed using the Lowry method (Bio-rad, 5000111). Electrophoresis was run using a bis-tris gel (Thermo Fisher, NW04122BOX), protein was transferred to a PVDF membrane (ThermoFisher, IB401001) using the iBlot2 transfer system (ThermoFisher, IB21001). Membrane was blocked using fluorescent blocking buffer (ThermoFisher, 37565). Membranes were incubated in anti-DNMT1 1/1,000 (Novus Biologicals, 60B1220.1), anti-survivin 1/200 (Abcam, ab469), anti-IRF5 1/500 (Cusabio, CSB-PA011820LA01HU), or anti-GAPDH 1/1,000 (Santa Cruz Biotech, sc-32233) overnight followed by incubation in fluorescent secondary antibody (ThermoFisher, A21057 or A11369) for 1 h at room temperature. Signal was visualized using the iBright imaging system (ThermoFisher, FL1500).

### Tumor Cell Implantation and Measurements

KPT277 cells were implanted subcutaneously into the left flank of 8–9 week old C57Bl/6 mice in sterile HBSS without calcium and magnesium (Hyclone, SH30588.01) at a total of 2 million cells per mouse. This same experiment was also done in NSG immunocompromised mice (Jackson labs, 005557). Once tumors reached an average volume of 100 mm^3^ (day 6), treatments with either DMSO or 5-Azacytidine began via intraperitoneal injection. 5-Azacytidine was used at a concentration of 1 mg/kg in a total volume of 400 μl HBSS. DMSO concentration used per treatment in either 5-Azacytidine or DMSO treated mice was ~0.5%. Tumors were measured three times weekly with calipers and volume was calculated using the equation: (width^2^ × length)/2. Mice were treated and handled humanely according to approved IACUC protocol #17128. Mice were euthanized once tumors reached a maximum length of 15 mm.

### Flow Cytometry

#### Cell Lines

1 × 10^6^ live cells were counted using trypan blue and first stained with a fixable viability dye (eBiosciences, 65-0866-14) for 30 min at 4°C. Cells were washed in flow wash buffer (PBS with 0.1% sodium azide and 1% FBS) and stained with surface antibodies for 40 min at 4°C. Cells were washed in flow buffer and fixed in flow buffer plus 1% PFA before filtering through 40 μM mesh strainer/tube (BD Biosciences). Flow cytometry was performed on the BD FACSCelesta cytometer and data was analyzed using FlowJo Version 10 (Becton, Dickinson & Co.).

#### Tumors

Tumors were excised and digested mechanically by mincing with a sterile scalpel before digestion in 1 mg/ml collagenase I (Sigma, C5138) plus 1% FBS for 1.5 h shaking at 200 rpm in a 37°C incubator. Dissociated tumor cells were spun at 450 g for 10 min and filtered through a 70 μm strainer. These cells were stained for flow cytometry following the above protocol.

#### Flow Cytometry Antibodies Used

BV650 rat anti-mouse CD45 #563410 (BD Horizon), APC rat anti-mouse CD8a #553035, PE anti-mouse CD152 (CTLA-4) # 553720 (BD Pharmingen), Alexa Fluor 700 anti-mouse NK1.1 #56-5941-82, eFluor450 anti-mouse CD279 (PD-1) #48-9985-82, PerCP-Cy5.5 anti-mouse CD4 #45-0042-80 (Invitrogen).

### *Ex-vivo* Cytotoxicity Assay

KPT277 cells were plated to 40% confluency in a 96 well plate and treated for 96 h in full media with DMSO or Aza. Splenocytes were isolated from female mice harboring subcutaneous KPT277 tumors and incubated in RPMI 1640 media (Corning, 10-040-CV) with 10% FBS, penicillin/streptomycin, L-glutamine, and 10% T-STIM (Corning, 354115). Media containing DMSO or Aza was removed from KPT277 cells and splenocytes in media were added 100:1 based on final cell counts from KPT277 wells. Co-incubation occurred for 24 h. After 24 h, media plus splenocytes was removed and wells were washed carefully with PBS. A standard MTT assay was done on KPT277 wells to determine viability compared to wells without splenocytes.

### Tissue Immunohistochemistry (IHC)

Briefly, tissue samples were sectioned at a thickness of 5 μm. Deparaffinization, rehydration, endogenous peroxidase activity inhibition, and antigen retrieval were all performed on the Ventana Discovery Ultra IHC (Roche Diagnostics) automated stainer. Slides were then incubated with primary antibodies, followed by DISCOVERY HQ and DISCOVERY HQ-HRP system, visualized with ChromoMap DAB detection Kit (Ventana). The slides were then counterstained with haematoxylin (Ventana) and coverslipped. CD4 rabbit monoclonal 1:100, CD8a rabbit monoclonal 1:100, and CD11C rabbit monoclonal 1:100 were all purchased from Cell Signaling and F4/80 rat monoclonal 1:200 from Bio-Rad.

## Results

### Transition From ADM to PDAC Is Characterized by Significant Increases in TE Expression and Tumor Associated Antigens

Previous studies have characterized the frequency and types of TEs in various cancers at early- and late-stage development (35, 36), however, the evolution of TE signatures from pre-malignancy to malignancy is virtually unknown. This is important as unique TE signatures in pre-malignant tissues that contribute to or are predictive of malignant transformation may be useful in the design of early diagnostic or therapeutic strategies (37). To this end, we determined the frequency and types of TEs expressed during pre-malignant (ADM) and malignant (PDAC) stages of pancreatic cancer utilizing an inducible model which restricts the oncogenic alleles *Kras*^*G*12*D*^ and *Trp53*^*R*270*H*^ to pancreatic, acinar-specific expression through a tamoxifen-inducible Ptfa1-Cre promoter (38, 39). Intraperitoneal administration of tamoxifen in 4–5 week old mice consistently results in a steady chronologic progression to PDAC that is equivalent to that observed in humans: ADM > pancreatic intraepithelial neoplasia (PanIN) > PDAC > liver and lung metastases (40). In addition to histopathology by hematoxylin/eosin staining, we confirmed the identity of premalignant and malignant lesions by immunofluorescent staining (Supplementary Figure 1). Healthy pancreatic (HP) acinar cells are positive for Cpa1, while being negative for the ductal markers CK19 and Sox9 (Supplementary Figure 1A). In contrast, lesions representative of ADM are Cpa1^+^Sox9^lo^CK19^lo^ (Supplementary Figure 1B), while PanIN and PDAC lesions transition to a more ductal phenotype (Cpa1^lo/−^Sox9^+^CK19^+^) (Supplementary Figures 1C,D) (41). Thus, using this model, we would be able to determine TE signatures at the various stages of PDAC development.

**Figure 1:**
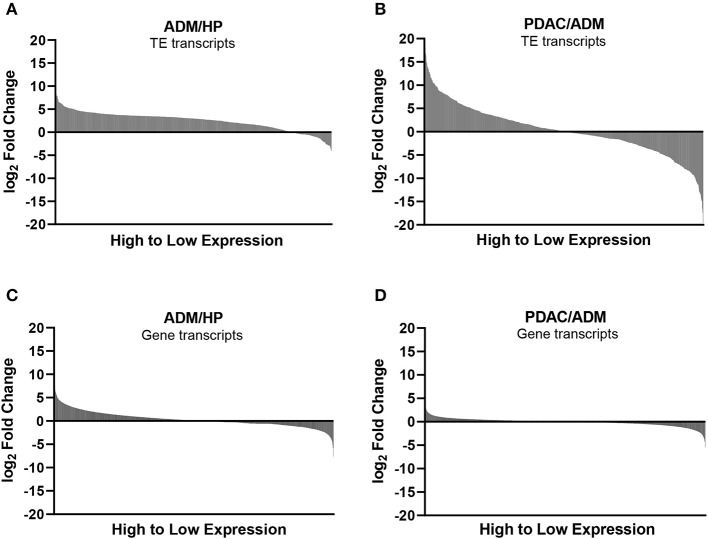
Expression of transposable elements (TEs) and non-TE, gene-encoded transcripts during transition to malignancy. **(A)** Waterfall plots of TE expression, using RNA-seq, during the transition from healthy pancreas (HP) to acinar-to-ductal metaplasia (ADM) (pre-malignant) and **(B)** from ADM to pancreatic ductal adenocarcinoma (PDAC) (malignant). **(C)** Waterfall plots of non-TE, protein coding gene transcripts displayed in order from highest to lowest fold change for ADM/HP and **(D)** PDAC/ADM.

Mouse gene and TE transcript profiles for tissues representing HP, ADM, and PDAC were generated through laser capture microdissection followed by RNA sequencing (RNA-Seq). TE sequence expression was mapped to the RepBase mouse transposon database and is represented as fold change from HP to ADM (ADM/HP) or ADM to PDAC (PDAC/ADM) with TEs organized by highest positive fold change to highest negative fold change (Figures 1A,B). The majority of TEs were significantly upregulated in ADM compared to HP, suggesting an immediate dysregulation of TE suppression coincident with transformation. Interestingly, during the transition from ADM to malignant PDAC, more drastic changes occur with some TEs remaining high, a few continuing to increase, and some decreasing back to HP equivalent expression (Supplementary Tables 1, 2). TEs that become increasingly expressed exclusively during the ADM to PDAC transition are listed in Table 1. Taken together, these data suggest that while premalignant stages are associated with global dysregulation (upregulation) of TEs, transition to malignant PDAC in this model is associated with augmented expression of only a distinct collection of TEs. This represents a unique TE signature that has not yet been described during malignant transformation in pancreatic cancer. In addition to analyzing the TE signature from pre-malignancy to malignancy in our PDAC model, we also analyzed the expression of protein-coding genes (Figures 1C,D). Transcripts of highly immunogenic TAA such as mesothelin and mucins (42, 43) were significantly upregulated during the transition from HP to ADM (and remained high in PDAC) or were unchanged during the transition from HP to ADM but increased in their expression during the transition from ADM to PDAC (Table 2).

**Table 1:** Fold change (FC) in significantly increased transposable elements (TE) during transition from acinar-to-ductal metaplasia (ADM) to pancreatic ductal adenocarcinoma (PDAC).

**Unique TE ID**	**Log2 FC^†^**	***p*-value^†^**
L1ME4c_3end|Eutheria	18.41	****
L1MB4_3end|Eutheria	18.22	****
Charlie19a|Mammalia	17.4	****
MLT1E2|Eutheria	17.36	****
MER63B|Eutheria	15.88	****
RMER3D3|Muridae	15.71	****
L1M2_orf2|Eutheria	15.2	****
MamGypLTR1c|Mammalia	15.06	****
MLT2F|Eutheria	14.41	****
L1MCb_5end|Eutheria	14.39	****
LTR16B1|Eutheria	14.21	****
MER66C|Eutheria	14.16	****
Tigger9b|Eutheria	14.11	****
LTR16E2|Eutheria	13.73	****
MER76|Eutheria	13.71	****
MER96|Eutheria	13.16	****
MER97a|Eutheria	12.8	****
RLTR16C_MM|Mus_musculus	12.64	****
Charlie1a|Eutheria	12.32	****
Arthur1C|Eutheria	11.8	****
LTR34|Eutheria	11.77	****
ERV3-16A3_LTR|Eutheria	11.72	****
RMER3D2|Muridae	11.52	****
MER121|Mammalia	11.28	****
C573_MM|Mus_mouse_genus	11.24	****
L1ME3C_3end|Eutheria	11.18	****
MER66B|Eutheria	11.07	****
MER58B|Eutheria	10.99	****
MER110A|Eutheria	10.94	****
RMER16C|Muridae	10.55	****
RLTR30|Muridae	10.39	****
L2a_3end|Mammalia	10.21	****
LTR44|Eutheria	10.17	****
LTR102_Mam|Mammalia	9.999	****
LTR41B|Eutheria	9.866	****
RLTR9C|Mus_mouse_genus	9.812	****
MLT1J2|Eutheria	9.79	****
MER5A1|Eutheria	9.772	****
LTR52|Eutheria	9.771	****
MER34A|Eutheria	9.759	****
RLTR12H|Muridae	9.562	****
LTR81C|Mammalia	9.316	****
Tigger5b|Eutheria	9.226	****
MER44C|Eutheria	9.189	****
MER66A|Eutheria	9.035	****
L1M3e_5end|Eutheria	8.959	****
Charlie7a|Eutheria	8.867	****
RLTR12C|Muridae	8.834	****
MER92C|Eutheria	8.757	****
RLTR9E|Mus_mouse_genus	8.744	****
L1ME3D_3end|Eutheria	8.7	****
Charlie9|Eutheria	8.67	****
L1M7_5end|Mammalia	8.594	****
LTR103b_Mam|Mammalia	8.59	****
MTD|Rodentia	8.571	****
MER102a|Eutheria	8.552	****
L1ME1_3end|Eutheria	8.506	****
MamTip1|Mammalia	8.445	****
MER67D|Eutheria	8.44	****
L1MA7_3end|Eutheria	8.318	****
ERVL-B4|Eutheria	8.227	****
MER106B|Eutheria	8.22	****
RMER16B3|Muridae	8.175	****
L1MEg_5end|Mammalia	8.125	****
Charlie2a|Eutheria	8.091	****
LTR81A|Mammalia	8.056	****
MER97b|Eutheria	8.052	****
MER101B|Eutheria	8.049	****
FordPrefect_a|Eutheria	8.037	****
MLT1A|Eutheria	8.005	****
ORR1F|Murinae	7.858	****
L1MEh_5end|Mammalia	7.781	****
Tigger17c|Eutheria	7.777	****
Charlie15a|Mammalia	7.741	****
RLTR20B5_MM|Mus_musculus	7.566	****
MER34|Eutheria	7.448	****
Charlie10b|Eutheria	7.437	****
LTR105_Mam|Mammalia	7.43	****
MamRep564|Eutheria	7.417	****
Tigger8|Eutheria	7.374	****
L1ME4a_3end|Eutheria	7.357	****
L1M4b_5end|Eutheria	7.312	****
L1M4b_5end|Eutheria	7.312	****
MER73|Eutheria	7.267	****
LTR28|Eutheria	7.23	****
Charlie15b|Mammalia	7.213	****
MER3|Eutheria	7.189	****
Charlie18a|Mammalia	7.126	****
Tigger12|Mammalia	7.05	****
URR1B|Muridae	7.047	****
LTR9|Eutheria	6.938	****
MLT2B3|Eutheria	6.824	****
MER57D|Eutheria	6.671	****
LTR33C|Eutheria	6.604	****
L1M6_5end|Mammalia	6.336	****
Charlie5|Eutheria	6.298	****
MER106A|Eutheria	6.28	****
L1MA4A_3end|Eutheria	6.26	****
L1MA4_3end|Eutheria	6.248	****
MER74C|Eutheria	6.207	****
MER58C|Eutheria	6.172	****
MER74B|Eutheria	6.056	****
LTR75_1|Eutheria	5.892	****
Tigger9a|Mammalia	5.884	****
L1M3b_5end|Eutheria	5.86	****
RLTR11C_MM|Mus_musculus	5.856	****
MER45A|Eutheria	5.848	****
MIR|Mammalia	5.719	***
L1ME3_3end|Eutheria	5.644	***
L1MC3_3end|Eutheria	5.475	***
FordPrefect|Eutheria	5.471	***
Tigger17a|Mammalia	5.418	***
RMER12C|Muridae	5.41	***
RLTR12B|Muridae	5.387	***
L1ME2_3end|Eutheria	5.381	***
LTR64|Eutheria	5.381	***
LTR78B|Eutheria	5.381	***
MER103C|Eutheria	5.372	***
MamGyp-int|Mammalia	5.345	**
RMER15-int|Muridae	5.315	**
MER112|Eutheria	5.255	*
MER91B|Eutheria_Mammalia	5.247	**
MER5A|Eutheria_Mammalia	5.241	**
MER33|Eutheria	5.219	**
RMER3D1|Muridae	5.171	**
MLT1G1|Eutheria	5.147	**
CYRA11_Mm|Mus_mouse_genus	5.14	**
MER58A|Eutheria	5.134	**
LTR78|Mammalia	5.091	**
L1MA5A_3end|Eutheria	5.089	**
MTE2a|Rodentia	5.084	**
L1M5_orf2|Eutheria	5.069	**
MER113B|Eutheria	5.044	**
LTR55|Eutheria	4.976	**
L1MB8_3end|Eutheria	4.963	**
LTR37A|Eutheria	4.912	**
MER34A1|Eutheria	4.896	**
MLTR73|Murinae	4.845	**
L1_Mur3_orf2|Muridae	4.824	*
RMER16-int|Muridae	4.821	*
LTR73|Eutheria	4.74	*
RLTR10B|Mus_mouse_genus	4.732	*
MER45R|Eutheria	4.724	*
LTR59|Eutheria	4.697	*
MER105|Eutheria	4.665	*
ORR1G|Murinae	4.663	*
MER67C|Eutheria	4.638	*
LTR48B|Eutheria	4.593	*
RLTR13C2|Mus_mouse_genus	4.585	*
MER65D|Eutheria	4.467	*

† *Fold change (FC) calculated using raw read values per TE*.

p-values adjusted using Sidak's multiple comparisons test,

* *p < 0.05*,

** *p < 0.01*,

*** *p < 0.001*,

**** *p < 0.0001*.

**Table 2:** Gene-encoded, tumor-associated antigens significantly upregulated during transition from healthy pancreas (HP) to acinar-to-ductal metaplasia (ADM) and ADM to pancreatic ductal adenocarcinoma (PDAC).

**Gene Name**	**Protein**	**Fold Change (Log_**2**_)**	***p*-value^†^**
**ADM/HP**			
Muc6	Mucin	10.303	5.1e-35
Muc5ac	Mucin	9.608	2.51e-30
Shh	Sonic hedgehog	7.816	2.21e-12
Tns4	Tensin	7.687	4.03e-45
Muc13	Mucin	6.052	4.10e-08
Psca	Prostate stem-cell antigen	5.901	4.04e-05
Muc4	Mucin	5.683	1.38e-10
Muc3a	Mucin	4.572	9.70e-08
Ccnb1	Cyclin-B1	4.510	3.62e-07
Gli1	Glioma-associated oncogene	3.703	1.31e-28
**PDAC/ADM**			
Muc20	Mucin	3.551	4.26e-32
Msln	Mesothelin	3.079	9.13e-20

† *p-values adjusted for multiple comparisons using the Benjamini-Hochberg procedure*.

### Upregulation of TE and TAA Expression in PDAC Coincides With Downregulation of Gene Transcripts for Antigen Presentation, T Cell Recruitment and Anti-viral Immunity

Transposable elements and certain TAAs drive tumor progression and contribute to the metastatic potential of various cancer types (3, 44, 45), which is likely the reason that increased expression coincides with transition to malignancy in our autochthonous PDAC model (Tables 1, 2). However, the observation that highly immunogenic TEs and TAAs significantly increase during transformation to PDAC, but do not cause a significant anti-tumor response to prevent progression and growth, would suggest that components involved in anti-viral immunity and/or antigen-presentation are compromised during this transition. In fact, recent studies in immunocompetent melanoma, ovarian, colorectal and breast cancer models (14, 15, 25) have shown that activation (i.e., upregulated expression) of TEs in conjunction with increased, innate type I interferon signaling and MHC class I expression, through DNMTi treatment, sensitizes tumor cells to immune attack, which contributes to dramatically decreased tumor growth. These data would suggest that the benefit of anti-TAA or -TE immunity to control tumor growth outweighs their tumor promoting activity.

**Figure 2:**
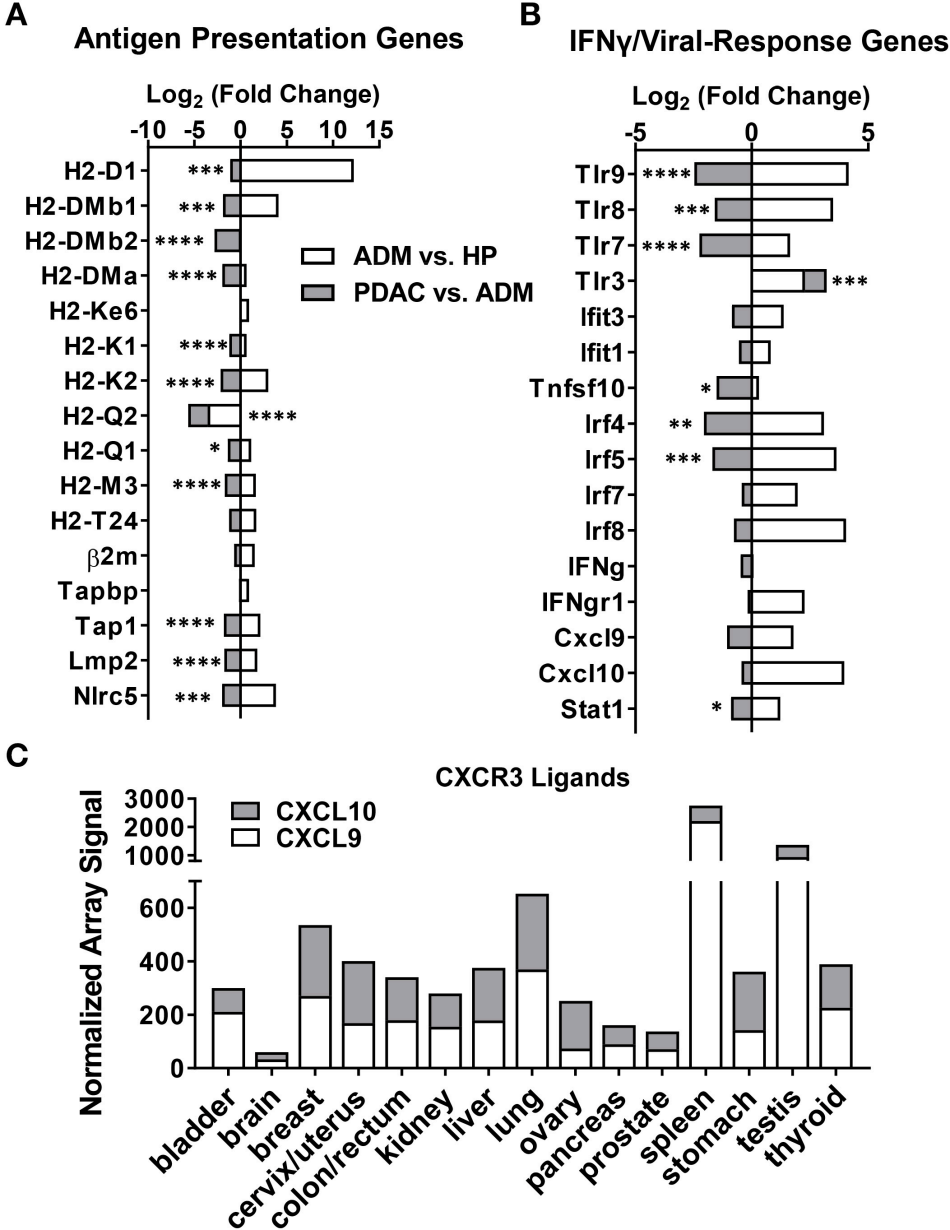
Antigen presentation and viral response-associated genes decrease following malignant transformation to PDAC. Log_2_ fold-change in gene expression from RNA-seq analysis, calculated as the ratio of ADM fold-change over HP (white) or PDAC fold-change over ADM (gray) (*n* = 3 mice per group). **(A)** Fold-change in antigen presentation-related genes. **(B)** Fold-change in IFN-γ and innate immune-related genes. **(C)** Human microarray dataset (E-GEOD-2019) was analyzed for signal for CXCL9 and CXCL10 in various cancer types. *p*-values shown are for significance in fold change of PDAC over ADM. *p < 0.05, **p < 0.01, ***p < 0.001, ****p < 0.0001.

In support of this hypothesis, we found that major histocompatibility complexes (H2-D, H2-K, H2-Q) and associated antigen processing/presentation machinery (Tap, Nlrc5, Lmp2) were significantly downregulated during the transition from ADM to PDAC (Figure 2A). Concurrent upregulation of TAAs and TEs with downregulation of antigen processing/presentation highlights a critical escape mechanism in PDAC that may select for tumor-promoting gene expression and retrotranspositions while evading immune detection. This is further highlighted by the significant downregulation of genes involved in innate and adaptive anti-viral immune responses (which would be activated by dsDNA intermediates from TEs) including NFkB-associated genes (Irf4, Irf5), toll-like receptors (TLRs), and Stat1, which is induced by IFN-γ (Figure 2B). Although not significant, Cxcr3 ligands (Cxcl9, Cxcl10), IFN-γ and other interferon-inducible proteins (Ifit1 and Ifit3) were also downregulated. Using gene expression array data from the Expression Project for Oncology database (E-GEOD-2109), we observed strikingly low expression of CXCL9 and CXCL10 in human PDAC compared to other cancer types (Figure 2C), possibly indicating escape from anti- tumor surveillance by downregulation of chemokines that would normally recruit NK and T cells. Together these data suggest that while TE and TAA upregulation is correlated with progression from ADM to PDAC, detection of antigen-expressing malignant cells during this time is averted by the coincident downregulation of antigen presentation, T cell recruitment, and anti-viral immunity.

### Low Dose Aza Augments Expression of T Cell Chemokines and Antigen Processing/Presentation Machinery in Murine and Human PDAC Cell Lines

The KPT277 cell line, which was derived from a spontaneous PDAC tumor isolated from a KPT mouse (described in Supplementary Figure 1), was used to evaluate changes in the expression of T cell chemokines and antigen processing/presentation machinery following Aza treatment. For additional comparison, we also evaluated changes in the murine PDAC cell line Pan02 and human PDAC cell lines PANC-1, MiaPaCa-2 and Hs-766-T following Aza treatment. For these studies, we first determined an LD25 dose of Aza for each cell line *in vitro* by treating with increasing concentrations of Aza and then determining cell viability 96 h later. As expected, Aza decreased cell viability in all cell lines in a dose-dependent manner (Supplementary Figure 2A). The dose of Aza that resulted in ~25% and/or significantly decreased viability for each cell line was used for subsequent RNA expression studies. For KPT277 cells, the Aza dose chosen is able to significantly inhibit DNMT1 expression by 96 h of treatment without major loss in viability (Supplementary Figure 2B).

**Figure 3:**
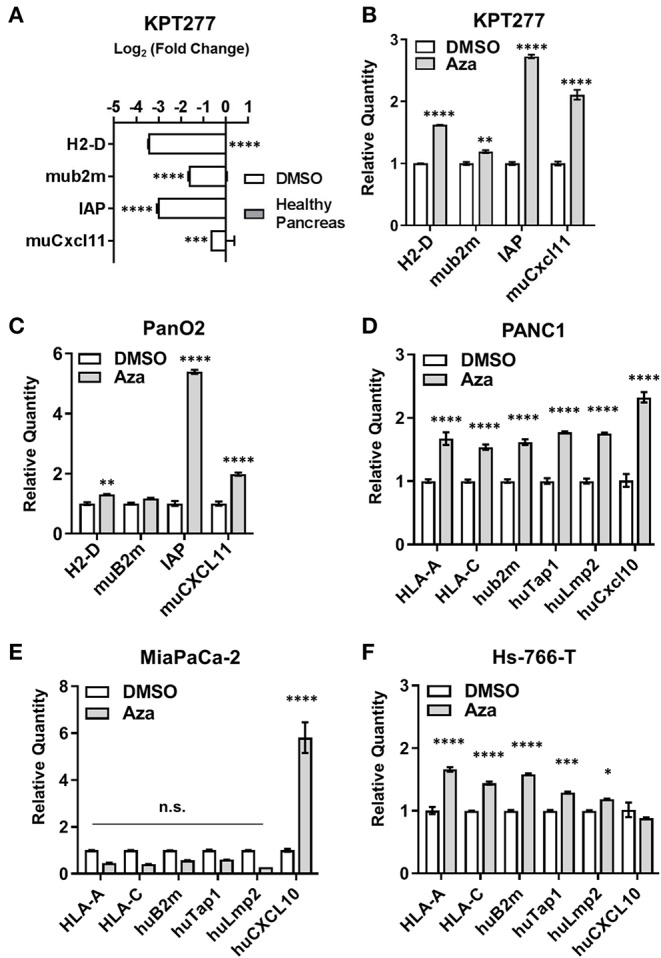
Aza treatment augments antigen presentation- and T cell recruitment-related gene expression in mouse and human PDAC cells. qRT-PCR was performed on total RNA to assess gene expression for **(A)** KPT277 cells treated with DMSO for 96 h *in vitro* vs. healthy pancreas from a C57Bl/6 mouse, and **(B)** KPT277 cells treated *in vitro* with DMSO or 10 μM Aza for 96 h. **(C)** qRT-PCR for the mouse PDAC cell line Pan02 treated *in vitro* with DMSO or 5 μM Aza. **(D–F)** Human PDAC cell lines treated *in vitro* with DMSO or Aza (100 μM for PANC1; 10 μM for MiaPaCa-2; and 50 μM for Hs-766-T) for 96 h. In **(A)**, bars represent Log base 2-fold change relative to “Healthy Pancreas.” *n* = 3 biological replicates. In **(B)** and **(C–F)**, bars represent quantity of transcripts relative to vehicle (DMSO) for each gene. *n* = 3 biological replicates. *p*-values were assessed by[[Inline Image]] two-way ANOVA followed by Sidak's multiple comparisons test. *p < 0.05, **p < 0.01, ***p < 0.001, ****p < 0.0001. n.s. = not significant.

Relative to healthy pancreas, untreated KPT277 cells show significant decreases in transcripts encoding antigen presentation machinery and T cell chemokines as determined by quantitative PCR (qPCR) (Figure 3A). QPCR analysis of KPT277 cells treated with the minimal effective dose of Aza revealed increased expression of antigen presentation/processing-associated transcripts and CXCR3 ligands (H2-D, muB2m, integrin-associated protein (IAP), and muCXCL11) (Figure 3B). Reprogramming of these gene transcripts was also confirmed for Pan02 following Aza treatment (Figure 3C). To evaluate changes in DNA methylation following Aza treatment of KPT cells, PCR was performed on a CpG island in the promoter region of IAP using primers specific to methylated or unmethylated DNA. Quantified PCR bands show that Aza treatment increased de-methylation of IAP over time, compared to vehicle-treated cells, indicating that increased expression of IAP after Aza treatment may be due to direct de-methylation of the promoter (Supplementary Figure 2C). Similar to murine cell lines, treatment of human PDAC cell lines with LD25 doses of Aza upregulated expression of HLA-A, HLA-C, huB2m, huTap1, huLmp2, and huCXCL10 compared to vehicle (DMSO) alone (Figures 3D–F). Not all changes were uniform in Aza-treated human cell lines. All cell lines, however, did show significant upregulation of one or more antigen presentation genes/CXCR3 ligands.

### Aza Treatment Induces Splenocyte Toxicity That Is Not Enhanced by PD-1 Checkpoint Blockade

**Figure 4:**
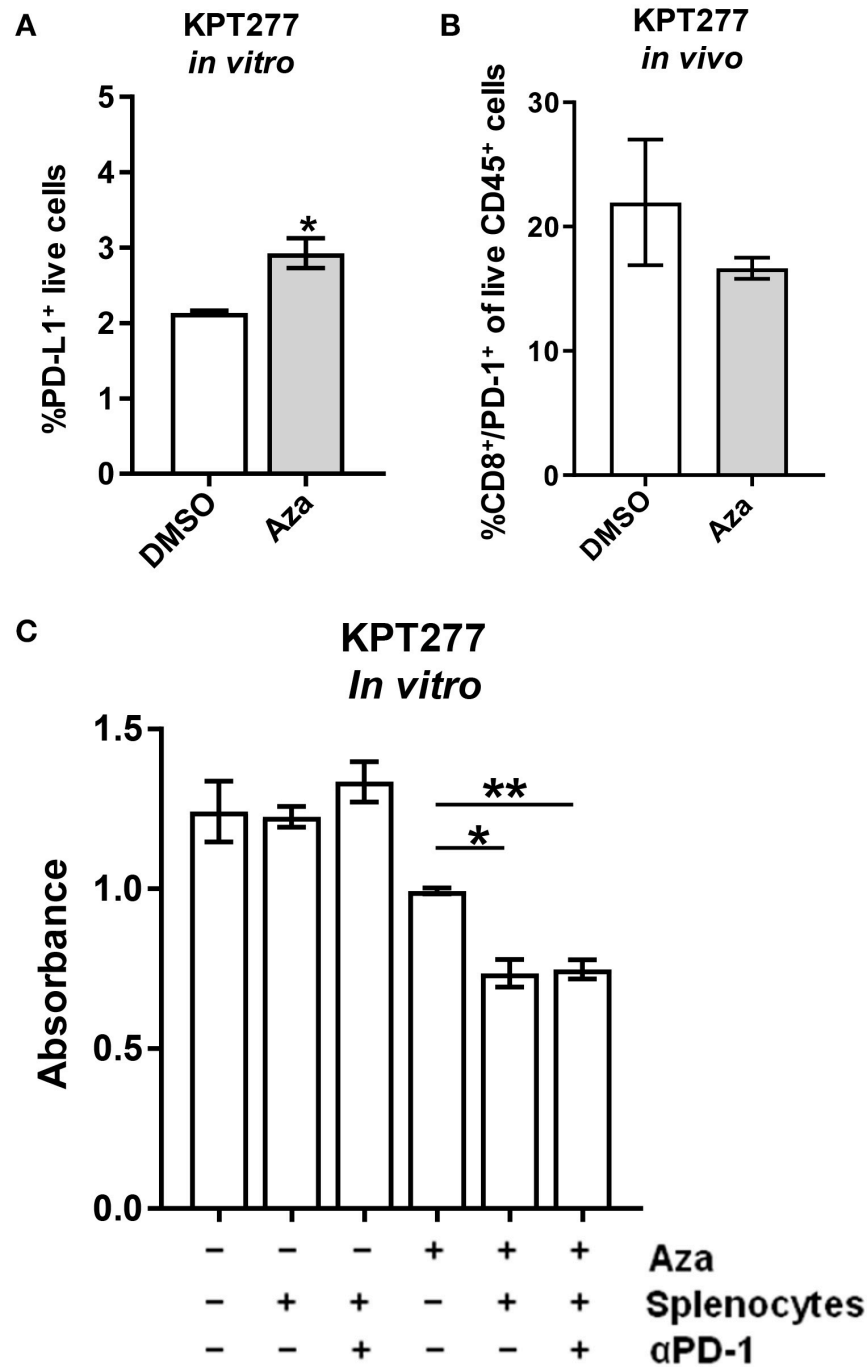
Aza treatment potentiates splenocyte killing of KPT277 cells that is not enhanced by PD-1 checkpoint blockade. **(A)** KPT277 cells were treated with Aza at 20 μM *in vitro* for 96 h and analyzed by flow cytometry using a fluorescent, fixable viability dye and an antibody recognizing PD-L1. Bars represent living, %PD-L1 positive cells out of total cells. *n* = 3 biological replicates. **(B)** KPT277 cells pre-treated for 72 h with DMSO or Aza (20 μM) were implanted in mice subcutaneously for 48 h. Tumors were then removed, digested, and analyzed by flow cytometry for PD-1, CD-8, and CD45 expression as well as live/dead cells using a fixable viability dye. Bars represent the percent of PD-1^+^/CD8^+^ cells out of live, CD45^+^ cells. *N* = 2 tumors per group. **(C)** KPT277 cells treated with DMSO or 20 μM Aza (72 h) were incubated with or without splenocytes (100:1) from an Aza pre-treated (10 μM for 96 h), KPT277 tumor-burdened mouse. Splenocytes pre-treated with anti-PD-1 antibody (2.5 μg/mL) *in vitro* for 1 h are indicated. KPT277 cell viability after 24 h of co-incubation was assayed by MTT. Bars represent absorbance values at 595 nm. *n* = 3 biological replicates. Error bars represent SEM. *p*-values were calculated using an unpaired *t*-test. *p < 0.05 and **p < 0.01.

Previous studies using DNMTis to increase the immunogenicity of tumor cells have reported a concomitant increase in the expression of immune checkpoint proteins, such as CTLA-4 and/or PD-L1 on the surface of treated cells, necessitating combination treatment with checkpoint immunotherapy (14, 46). To explore whether this therapeutic combination may be necessary in PDAC, we treated KPT277 cells in culture with Aza for 96 h to asses PD-L1 surface expression by flow cytometry. PD-L1 expression was significantly increased on Aza-treated cells, although the overall percentage of PD-L1 positive live cells was low in both groups (<5%) (Figure 4A). To assess whether epigenetic alterations in Aza-treated tumor cells might increase PD-1 expression on T-cells *in vivo*, we implanted KPT277 cells pre-treated with Aza subcutaneously into mice. Forty-eight hours post-implantation, tumors were removed and digested into single cell suspensions for analysis by flow cytometry. Figure 4B shows no significant increase in CD8/PD-1 double-positive cells from the CD45 positive tumor fraction, indicating that Aza-treated KPT277 cells do not induce PD-1 expression in tumor-associated CD8 T cells.

To evaluate whether Aza treatment of KPT277 cells is able to stimulate splenocyte anti-tumor cytotoxicity and whether PD-1/PD-L1 expression might interfere, DMSO or Aza-treated KPT277 cells were co-incubated with splenocytes from mice implanted with KPT277 tumors. Co-incubation of DMSO-treated KPT277 cells with splenocytes did not change tumor cell viability compared to DMSO-treated KPT277 cells not incubated with splenocytes (Figure 4C). However, Aza-treated KPT277 cells experienced an ~25% reduction in viability after co-incubation with splenocytes compared to incubation without splenocytes. Splenocytes pre-treated with anti-PD-1 antibody did not cause greater decreases in viability compared to splenocytes pre-treated with isotype control antibody. These results indicate that Aza treatment alone increases the immunogenicity of KPT277 cells that allows for enhanced killing by splenocytes and that combination treatment with PD-1 checkpoint inhibition does not provide additional benefit.

### Aza Treatment Controls KPT277 Tumor Growth via an Immune-Dependent Mechanism

**Figure 5:**
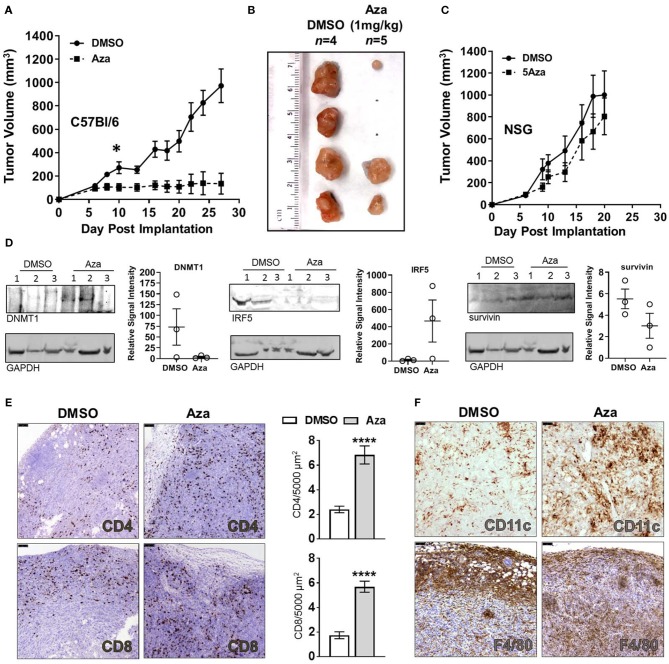
Aza treatment controls PDAC tumor growth through an immune-dependent mechanism and induces significant infiltration by CD4^+^ and CD8^+^ T cells. **(A)** KPT277 tumor growth curves from immunocompetent C57Bl//6 mice treated with vehicle (DMSO) or 1 mg/kg 5-Azacytidine (Aza) once weekly. Measurements were taken three times weekly. Curves represent mean measurements for *n* = 4 mice in the DMSO group and *n* = 5 in Aza-treated group. **(B)** Tumors after removal from C57Bl/6 mice euthanized on Day 28. Ruler (cm) shows approximate tumor lengths. **(C)** KPT277 tumor growth curves from immunocompromised NSG mice treated with vehicle (DMSO) or 1 mg/kg 5-Azacytidine (Aza) once weekly. Measurements were taken three times weekly. Curves represent mean measurements for *n* = 5 mice in the DMSO group and *n* = 6 in Aza-treated group. Error bars represent standard error of the mean (SEM). *p < 0.05, *t*-test comparing data between groups on Day 10. **(D)** Western blots of tumor lysates (*n* = 3 per group) from Day 8 Aza and DMSO-treated tumors (48 h after first treatment) using anti-DNMT1, anti-IRF5, and anti-survivin antibodies. GAPDH is shown as a loading control. DNMT1 and survivin were assayed using the same blot. Relative quantifications of band intensity normalized to GAPDH are shown to the right of blots (individual values plotted, error bars = SEM). Increased frequency and infiltration of **(E)** CD4^+^ and CD8^+^ T cells, and **(F)** CD11c^+^ and F4/80^+^ cells as determined by IHC in tumor tissue fixed 48 h after initial dosing with DMSO or Aza. Scale bars = 75 μm. Dab signal (brown) indicates CD4+, CD8+, CD11c+, or F4/80+ staining. Nuclei were counterstained with hematoxylin. Positive-staining cells were enumerated for 30–40 fields approximating the entire tumor (*n* = 2 tumors DMSO, *n* = 3 tumors Aza) and are presented as cells per 5,000 μm^2^ tumor area (bar graph). Error bars represent SEM. ****p < 0.0001, Mann-Whitney test.

Considering the increased cytotoxicity of splenocytes toward Aza-treated KPT277 cells *in vitro*, we hypothesized that therapeutic treatment of mice using Aza would have efficacy against KPT277 tumors *in vivo*. To test this, immunocompetent mice were implanted with KPT277 cells subcutaneously and tumors were allowed to reach an average volume of 100 mm^3^ prior to treatment (day 6). Treatment with vehicle (DMSO) or Aza (1 mg/kg) starting on day 6 was repeated once weekly until tumors in the control group (DMSO) reached the maximum allowed size (15 mm in diameter, ~27 days post-implantation). Tumor growth curves revealed a striking difference between groups receiving Aza treatment vs. DMSO control (Figure 5A, Supplementary Figures 3A,B). For tumors treated with Aza, the average tumor volume remained unchanged following the first dose, indicating immediate and persistent control of tumor growth compared to tumors from DMSO-treated mice. Total regression of ≥40% of Aza-treated tumors by the end of the experiment indicates that Aza is capable of eliminating KPT277 tumors after repeated treatment (Figure 5B, Supplementary Figure 3C).

To determine whether tumor growth control by Aza treatment involves anti-tumor immunity or is due to direct toxicity by Aza, we performed identical KPT277 tumor treatment studies in immunodeficient NOD-*scid* IL2Rgamma^null^ (NSG) mice, which lack fully functional antigen presenting cells, natural killer cells and T and B cells (47). In NSG mice, we found no significant difference in KPT277 tumor growth between DMSO- and Aza-treated groups (Figure 5C), indicating that the decreased tumor growth observed in immunocompetent C57Bl/6 mice treated with Aza is due to a mechanism involving anti-tumor immunity and is not a direct, toxic effect.

### Aza-Treated Tumors Undergoing Tumor Control Show Greater Immune Cell Infiltration

To determine the immediate effects contributing to tumor control in Aza-treated, immunocompetent mice, tumors from a subset of mice were excised 24 or 48 h after the first Aza or vehicle treatment and examined for expression of antigen presentation-related genes by qPCR and western blot, as well as immune infiltrates by immunohistochemistry (IHC). Twenty-four hours post-initial treatment, Aza-treated tumors tended toward higher expression of Cxcl11 with no change in IAP (Supplementary Figure 4A). By 48 h, Aza-treated tumors showed no change for either target (Supplementary Figure 4B). Contamination by other cell types including immune cells and stromal cells, or the timing in which these changes occur, may have obscured increased expression in these genes after Aza treatment. Protein lysates from 48 h-treated Aza tumors showed decreased expression of DNMT1, consistent with the Aza mechanism of action, as well as increased expression of interferon regulatory factor 5 (IRF5), which in humans is a target of onco-exaptation by ERVs (48) as well as a downstream regulator of the innate immune response to dsDNA subsequent to TLR activation (49) (Figure 5D). Also observed in Aza-treated tumors was decreased expression of the anti-apoptotic tumor marker survivin, which is consistent with decreased tumor growth and regression seen in long-term Aza-treated mice (Figure 5A). Tumors showed high variability for all targets assayed, reminiscent of variability in tumor regression seen during Aza treatment. However, ≥66% of the Aza-treated tumors showed decreased DNMT1, increased IRF5 and decreased survivin, which is in line with the larger proportion of mice responding to Aza treatment (Figure 5B, Supplementary Figure 3B).

Interestingly, H&E staining of tumor tissue revealed greater intratumoral density of nuclei in Aza-treated tumors compared to DMSO treatment (Supplementary Figures 4C,D). Through immunohistochemistry (IHC) using specific antibodies for CD4 and CD8, T cells were located throughout Aza-treated tumor tissue within 48 h after receiving initial treatment (Figure 5E). A significantly lower frequency of T cells was present in tumors from DMSO-treated mice (*p* < 0.0001, Mann-Whitney), with a majority of the CD8^+^ T cell populations being restricted to the periphery of the tumor. These results suggest that Aza treatment induces changes to KPT277 tumors that cause greater recruitment of T cells into the interior of the tumor. In addition to adaptive immune cells, we observed overall increases in CD11c^+^ staining, representing dendritic cells (DC) and a small minority of natural killer (NK) cells, but quantification was not possible due to diffuse staining and cell clustering (Figure 5F, upper panels). F4/80^+^ cells (macrophages) showed a pattern of infiltration similar to that of CD8^+^ cells, wherein DMSO-treated mice show F4/80^+^ clustering at the periphery of tumor tissue and Aza-treated mice show more diffuse staining throughout the interior of tumors (Figure 5F, lower panels). Endpoint analysis of tumors from DMSO- and Aza-treated groups by flow cytometry revealed that CD8^+^ T cells were no longer elevated in uncontrolled or regressing Aza-treated tumors compared to vehicle, while the increased presence of CD4^+^ T cells could be observed in the regressing tumor, but not in the uncontrolled Aza-treated tumor (Supplementary Figures 3B, 4E,F). Moreover, expression of checkpoint molecules PD-1 or CTLA-4 was unchanged between Aza- and DMSO-treated groups. Altogether, these data strongly suggest that adaptive immune cells and professional antigen-presenting cells play an important role in control of KPT277 tumor growth immediately after Aza treatment, and that this results from epigenetic reprogramming conducive for eliciting anti-tumor immune responses.

## Discussion

In an autochthonous model of PDAC, we observed upregulation of several TEs and TAAs during malignant transformation that was concurrent with a downregulation of genes important for anti-tumor immune surveillance. Rescued (increased) expression of antigen presentation genes and T cell chemokines was possible with Aza treatment, suggesting that a selection for hypermethylated “anti-tumor” immunity genes, which would allow for immune escape, likely occurs during tumorigenesis. Therapeutic Aza treatment decreased growth of implanted PDAC tumors by enhancing the anti-tumor immune response, as evidenced by inhibited tumor growth in immune-competent mice, but not in immune-deficient mice. This anti-tumor immune response following Aza treatment correlated with significantly increased intratumoral infiltration of CD4^+^ and CD8^+^ T cells as well as CD11c^+^ DC and F4/80^+^ macrophages. Since Aza treatment increased antigen presentation and viral response genes *in vitro*, it is likely that tumor growth is controlled directly by the mechanism of de-methylation of these genes. However, we have not ruled out the possibility that additional, favorable immune responses occur due to Aza action on gene subsets of immune or other stromal cells. Regardless, these data suggest that DNMTi treatment has the potential to increase the immunogenicity of PDAC tumors through induction of innate and adaptive immunity resulting in tumor regression.

Although our studies have highlighted a correlation between increased expression of various TE families and certain TAAs with decreased expression of IFN-γ response/antigen-presentation genes during malignant transformation, more mechanistic studies would be needed to pinpoint whether expression of TEs or TAAs specifically drives anti-tumor immunity after Aza treatment. For example, future studies would need to determine whether observed upregulated TAAs are processed and presented on the surface of tumor cells after treatment with Aza, and whether knockdown of these TAAs abrogates anti-tumor immunity. In addition, the locus at which a TE has inserted into the genome can cause varied effects on its transcription and/or reverse transcription. Whether TE expression is autonomous or part of a host gene transcript can affect the outcome of transcription (50). The transcription of a TE element as part of a long non-coding RNA (lncRNA) may even act like small interfering RNA (siRNA) to silence its own expression (51). Thus, a more detailed analysis mapping these upregulated TEs to their various loci would need to be achieved before any direct link can be made between TE family expression and the possibility of reverse transcription into dsDNA that is immunogenic.

Regardless of the exact antigen(s) driving anti-tumor immunity in our model, Aza treatment caused tumor regression through an immune-mediated mechanism. A single dose of Aza resulted in increased infiltration of both CD4^+^ and CD8^+^ T cells throughout tumor tissue compared to DMSO-treated tumors. Qualitatively, there also appears to be an increase in the infiltration of CD11c^+^ cells and greater intratumoral distribution of F4/80^+^ cells. It is well-known that CD8^+^ T cells enact cytotoxic mechanisms during clearance of virally-infected cells and cells presenting MHC class I restricted antigens. CD11c^+^ NK cells and CD4^+^ T cells have also been shown to be directly cytolytic toward virally-infected cells in addition to their role in coordinating and priming CD8^+^ T cell immunity (52, 53). Studies utilizing Aza combined with CpG1826 in a syngeneic lung cancer model showed that NK1.1 depletion greatly abrogated the efficacy of the combined treatment, as did CD8 depletion, illuminating the importance of both immune arms in tumor control (54). In addition, professional antigen presenting cells, such as DC and macrophages, are necessary for proper activation of cytotoxic CD8^+^ T cells both through classical and cross-presentation of antigen to naïve T cells (55) and cytokine secretion (56). Growth curves from Aza-treated tumors support the cooperation of both innate and adaptive immune subsets as tumor growth control was seen immediately after the first treatment, but full regression required multiple doses over time. The immediacy with which these immune cells increasingly infiltrated tumors after Aza-treatment could indicate that *in vivo* effects occur much faster than *in vitro* effects of Aza treatment, and quick fluxes in gene expression may explain why changes in genes of interest were hard to detect.

In addition to increasing T-cell chemokines and IAP expression *in vitro*, DNMTi treatment in PDAC cell lines increased expression of MHC class I molecules. Increasing expression of MHC class I and other antigen-presentation machinery in tumors is of general interest and a critical unmet need, as decreased antigen presentation significantly reduces the effectiveness of many immunotherapeutic strategies (57–59). Hypomethylating agents, such as Aza, have the potential to meet this need. In gene expression data from breast cancer patients with matched biopsies before and after treatment with Aza and entinostat, the treatment increased the expression of multiple HLA molecules in all five patients (25). If a DNMTi such as Aza were able to increase MHC class I presentation of tumor antigens or IFN-γ responses in human PDAC, it may promote more favorable responses to current immunotherapies that PDAC, in general, has been highly refractive to Pu et al. (60).

However, various studies have shown that global gene de-methylation is not without its problems. In immunocompetent mouse models for breast and ovarian cancer, treatment with DNMTi has been correlated with increases in PD-1 or CTLA-4 checkpoint proteins, necessitating combinatorial treatment with checkpoint inhibitors despite increased expression of ERVs, anti-viral response genes and antigen presentation machinery (14, 25, 61). In a lung tumor model, where cancers were driven by HPV16, IL-2 therapy in conjunction with Aza treatment caused dramatic CD8^+^ T cell infiltration, which was required for anti-tumor efficacy (54), similar to the latter results of our study. The observation that Aza treatment in our model enhanced CD4^+^ and CD8^+^ T cell infiltration and caused significant, and in some cases complete, tumor regression without the need for checkpoint blockade or additional therapies, indicates that PDAC patients may benefit from the use of DNMT inhibitors as single agents.

We have presented data showing that Aza treatment not only enhances the immunogenicity of PDAC tumors, but also increases intratumoral T cell infiltration and suppresses growth without the need for checkpoint blockade. Phase 1 clinical trials involving pancreatic cancer patients to evaluate the combination of a DNMTi with chemotherapy and/or PD-1/PD-L1 blockade are currently recruiting (NCT03257761, NCT01845805, NCT02959164). Our studies may provide direction for maximizing efficacy of DNMTi therapy in patients, and insight into the possible mechanisms behind DNMTi anti-cancer activity.

## Data Availability Statement

The datasets generated for this study can be found in the Gene Expression Omnibus, Accession Number GSE111540.

## Ethics Statement

The animal study was reviewed and approved by the Institution Animal Care and Use Committee (IACUC) at the City of Hope.

## Author Contributions

NE conceived and designed experiments, acquired and analyzed data, and authored the manuscript. EZ acquired and analyzed data, edited manuscript. BJ conceived and designed experiments, acquired, and analyzed data. DD conceived ideas for this project and designed the experiments. EM conceived and designed experiments and edited the manuscript.

### Conflict of Interest

The authors declare that the research was conducted in the absence of any commercial or financial relationships that could be construed as a potential conflict of interest.
