# Supplementary material for: 5-Azacytidine Potentiates Anti-tumor Immunity in a Model of Pancreatic Ductal Adenocarcinoma
Source: Front Immunol. 2020 Mar 31;11:538. doi: 10.3389/fimmu.2020.00538 (PMC7136411; doi:10.3389/fimmu.2020.00538)
Supplement: Supplementary file 1 [file Table_1.DOCX]

**Supplemental Table 1.** Mean differences in fold change in expression of transposable elements during transition from healthy pancreas (HP) to acinar-to-ductal metaplasia (ADM).

| **Unique TE ID** | **Fold Change** | **95.00% CI of diff.** | **Summary** | **Adjusted P Value^†^** |
| --- | --- | --- | --- | --- |
| **ADM/HP** | | | | |
| 4.5SRNA\|Rodentia | 4.185 | 0.7565 to 7.614 | *** | 0.0008 |
| Arthur1B\|Eutheria | 4.856 | 1.427 to 8.285 | **** | <0.0001 |
| B1_Mm\|Mus_mouse_genus | 3.555 | 0.1261 to 6.983 | * | 0.0266 |
| B1_Mur1\|Muridae | 3.521 | 0.09189 to 6.949 | * | 0.0317 |
| B1_Mur2\|Muridae | 3.537 | 0.1085 to 6.966 | * | 0.0291 |
| B1_Mur3\|Muridae | 3.432 | 0.003367 to 6.861 | * | 0.0492 |
| B1_Mus1\|Mus_mouse_genus | 3.515 | 0.08583 to 6.943 | * | 0.0327 |
| B1_Mus2\|Mus_mouse_genus | 3.573 | 0.1448 to 7.002 | * | 0.0242 |
| B1F2\|Muridae | 3.505 | 0.07654 to 6.934 | * | 0.0342 |
| B3A\|Muridae | 4.541 | 1.112 to 7.970 | **** | <0.0001 |
| B4A\|Rodentia | 3.67 | 0.2415 to 7.099 | * | 0.0146 |
| BGLII_B\|Muridae | 3.665 | 0.2367 to 7.094 | * | 0.015 |
| BGLII_Mur\|Murinae | 4.078 | 0.6489 to 7.506 | ** | 0.0015 |
| BGLII_Mus\|Mus_mouse_genus | 4.074 | 0.6451 to 7.502 | ** | 0.0015 |
| BGLII\|Mus_mouse_genus | 3.699 | 0.2701 to 7.127 | * | 0.0126 |
| Cheshire\|Eutheria | 5.404 | 1.975 to 8.833 | **** | <0.0001 |
| DNA1_Mam\|Eutheria | 4.84 | 1.411 to 8.269 | **** | <0.0001 |
| ERVB2_1A-I_MM\|Mus_musculus | 4.404 | 0.9750 to 7.832 | *** | 0.0002 |
| ERVB4_1B-I_MM\|Mus_musculus | 6.322 | 2.894 to 9.751 | **** | <0.0001 |
| ERVB4_1B-LTR_MM\|Mus_musculus | 4.506 | 1.077 to 7.935 | *** | 0.0001 |
| ERVB4_1-LTR_MM\|Mus_musculus | 3.808 | 0.3795 to 7.237 | ** | 0.0069 |
| ERVB4_2-I_MM\|Mus_musculus | 3.604 | 0.1750 to 7.032 | * | 0.0207 |
| ERVB4_2-LTR_MM\|Mus_musculus | 4.032 | 0.6031 to 7.460 | ** | 0.002 |
| ERVB5_2-LTR_MM\|Mus_musculus | 4.429 | 0.9999 to 7.857 | *** | 0.0002 |
| ERVB7_2B-LTR_MM\|Mus_musculus | 3.824 | 0.3956 to 7.253 | ** | 0.0064 |
| ERVB7_2-LTR_MM\|Mus_musculus | 3.708 | 0.2793 to 7.137 | * | 0.0119 |
| ERVB7_3-LTR_MM\|Mus_musculus | 5.2 | 1.771 to 8.628 | **** | <0.0001 |
| ERVB7_4-LTR_MM\|Mus_musculus | 4.607 | 1.179 to 8.036 | **** | <0.0001 |
| ERVL\|Eutheria | 3.555 | 0.1264 to 6.984 | * | 0.0266 |
| ETnERV\|Mus_musculus | 3.695 | 0.2664 to 7.124 | * | 0.0128 |
| ETnERV2\|Mus_mouse_genus | 3.896 | 0.4668 to 7.324 | ** | 0.0043 |
| ETnERV3\|Mus_mouse_genus | 3.976 | 0.5468 to 7.404 | ** | 0.0027 |
| GSAT_MM\|Mus_mouse_genus | 3.62 | 0.1911 to 7.049 | * | 0.019 |
| HERVL32\|Eutheria | 3.583 | 0.1544 to 7.012 | * | 0.023 |
| IAP1-MM_I\|Mus_musculus | 3.958 | 0.5289 to 7.386 | ** | 0.003 |
| IAP1-MM_LTR\|Mus_musculus | 5.909 | 2.481 to 9.338 | **** | <0.0001 |
| IAPA_MM\|Mus_musculus | 4.089 | 0.6601 to 7.517 | ** | 0.0014 |
| IAPEY3C_LTR\|Mus_musculus | 3.806 | 0.3773 to 7.235 | ** | 0.007 |
| IAPEY3-int\|Mus_mouse_genus | 3.667 | 0.2378 to 7.095 | * | 0.0149 |
| IAPEY4_I\|Mus_musculus | 4.701 | 1.272 to 8.130 | **** | <0.0001 |
| IAPEY4_LTR\|Mus_musculus | 4.223 | 0.7939 to 7.651 | *** | 0.0006 |
| IAPEY5_I\|Mus_musculus | 3.68 | 0.2516 to 7.109 | * | 0.0139 |
| IAPEY5_LTR\|Mus_musculus | 4.399 | 0.9705 to 7.828 | *** | 0.0002 |
| IAPEy-int\|Mus_mouse_genus | 3.476 | 0.04733 to 6.905 | * | 0.0396 |
| IAPEz-int\|Mus_mouse_genus | 3.648 | 0.2193 to 7.077 | * | 0.0164 |
| IAPLTR1_Mm\|Mus_mouse_genus | 3.614 | 0.1852 to 7.043 | * | 0.0196 |
| IAPLTR1a_Mm\|Mus_mouse_genus | 3.743 | 0.3144 to 7.172 | ** | 0.0099 |
| IAPLTR2_Mm\|Mus_mouse_genus | 3.937 | 0.5078 to 7.365 | ** | 0.0034 |
| IAPLTR2a\|Mus_mouse_genus | 3.888 | 0.4591 to 7.316 | ** | 0.0045 |
| IAPLTR2a2_Mm\|Mus_mouse_genus | 3.749 | 0.3201 to 7.178 | ** | 0.0096 |
| IAPLTR2b\|Mus_mouse_genus | 3.736 | 0.3075 to 7.165 | * | 0.0103 |
| IAPLTR4_I\|Mus_mouse_genus | 4.21 | 0.7817 to 7.639 | *** | 0.0007 |
| IAPLTR4\|Mus_mouse_genus | 4.931 | 1.502 to 8.360 | **** | <0.0001 |
| IMPB_01\|Mus_musculus | 3.564 | 0.1355 to 6.993 | * | 0.0254 |
| L1_Mm_orf2\|Mus_mouse_genus | 3.618 | 0.1897 to 7.047 | * | 0.0192 |
| L1_Mur1_5end\|Muridae | 3.794 | 0.3654 to 7.223 | ** | 0.0075 |
| L1_Mur1_orf2\|Muridae | 3.614 | 0.1851 to 7.042 | * | 0.0196 |
| L1_Mus1_3end\|Mus_mouse_genus | 3.565 | 0.1364 to 6.994 | * | 0.0253 |
| L1_Mus2_3end\|Mus_mouse_genus | 3.498 | 0.06892 to 6.926 | * | 0.0356 |
| L1_Mus3_5end\|Mus_mouse_genus | 3.916 | 0.4869 to 7.344 | ** | 0.0038 |
| L1_Mus3_orf2\|Mus_mouse_genus | 3.497 | 0.06806 to 6.925 | * | 0.0357 |
| L1_Mus4_3end\|Mus_mouse_genus | 3.483 | 0.05390 to 6.911 | * | 0.0383 |
| L1_Rod_5end\|Muridae | 5.765 | 2.336 to 9.193 | **** | <0.0001 |
| L1M2a1_5end\|Eutheria | 4.094 | 0.6656 to 7.523 | ** | 0.0014 |
| L1M3c_5end\|Eutheria | 3.844 | 0.4155 to 7.273 | ** | 0.0057 |
| L1M3de_5end\|Eutheria | 4.68 | 1.251 to 8.108 | **** | <0.0001 |
| L1M4_5end\|Eutheria | 4.792 | 1.363 to 8.221 | **** | <0.0001 |
| L1M4a1_5end\|Eutheria | 5.401 | 1.972 to 8.829 | **** | <0.0001 |
| L1M4a2_5end\|Eutheria | 3.703 | 0.2739 to 7.131 | * | 0.0123 |
| L1M4c_5end\|Eutheria | 3.722 | 0.2935 to 7.151 | * | 0.0111 |
| L1MA10_3end\|Eutheria | 4.749 | 1.320 to 8.178 | **** | <0.0001 |
| L1MA6_3end\|Eutheria | 4.66 | 1.231 to 8.088 | **** | <0.0001 |
| L1MA8_3end\|Eutheria | 5.148 | 1.720 to 8.577 | **** | <0.0001 |
| L1MC4_5end\|Eutheria | 3.626 | 0.1969 to 7.054 | * | 0.0185 |
| L1MC4a_3end\|Eutheria | 4.143 | 0.7146 to 7.572 | ** | 0.001 |
| L1MC5_3end\|Eutheria | 4.359 | 0.9300 to 7.787 | *** | 0.0003 |
| L1Md_A_3end\|Mus_mouse_genus | 3.485 | 0.05627 to 6.914 | * | 0.0379 |
| L1Md_A_5end\|Mus_mouse_genus | 4.047 | 0.6185 to 7.476 | ** | 0.0018 |
| L1Md_F_5end\|Mus_mouse_genus | 3.633 | 0.2042 to 7.062 | * | 0.0178 |
| L1Md_F2_3end\|Mus_mouse_genus | 3.46 | 0.03117 to 6.889 | * | 0.0429 |
| L1Md_F3_3end\|Mus_mouse_genus | 3.472 | 0.04361 to 6.901 | * | 0.0403 |
| L1Md_Gf_5end\|Mus_mouse_genus | 4.073 | 0.6444 to 7.502 | ** | 0.0015 |
| L1MD_orf2\|Eutheria | 4.534 | 1.105 to 7.963 | **** | <0.0001 |
| L1Md_T_3end\|Mus_mouse_genus | 3.44 | 0.01161 to 6.869 | * | 0.0472 |
| L1MD2_3end\|Eutheria | 7.406 | 3.977 to 10.83 | **** | <0.0001 |
| L1ME3E_3end\|Eutheria | -3.975 | -7.404 to -0.5463 | ** | 0.0027 |
| L1ME3F_3end\|Eutheria | -3.501 | -6.929 to -0.07187 | * | 0.035 |
| L1ME5_3end\|Mammalia | 4.558 | 1.129 to 7.986 | **** | <0.0001 |
| L1MEa_5end\|Eutheria | 3.902 | 0.4731 to 7.331 | ** | 0.0041 |
| L1MEc_5end\|Eutheria | 3.852 | 0.4229 to 7.280 | ** | 0.0055 |
| L1VL1_5end\|Mus_mouse_genus | 3.605 | 0.1761 to 7.033 | * | 0.0206 |
| L1VL2_5end\|Mus_mouse_genus | 3.515 | 0.08675 to 6.944 | * | 0.0325 |
| L1VL4_5end\|Muridae | 3.619 | 0.1908 to 7.048 | * | 0.0191 |
| L2\|Mammalia | 4.455 | 1.026 to 7.883 | *** | 0.0002 |
| L3b_3end\|Mammalia | 5.441 | 2.012 to 8.870 | **** | <0.0001 |
| L4_A_Mam\|Mammalia | 3.466 | 0.03719 to 6.895 | * | 0.0416 |
| Looper\|Eutheria | 4.849 | 1.420 to 8.277 | **** | <0.0001 |
| LTR107_Mam\|Mammalia | 3.881 | 0.4524 to 7.310 | ** | 0.0046 |
| LTR33\|Eutheria | -3.584 | -7.013 to -0.1554 | * | 0.0229 |
| LTR37-int\|Eutheria | 3.549 | 0.1198 to 6.977 | * | 0.0275 |
| LTR41\|Eutheria | 3.474 | 0.04542 to 6.903 | * | 0.04 |
| LTR41C\|Eutheria | 5.262 | 1.834 to 8.691 | **** | <0.0001 |
| LTR68\|Eutheria | 4.375 | 0.9458 to 7.803 | *** | 0.0002 |
| LTR75B\|Eutheria | 3.612 | 0.1836 to 7.041 | * | 0.0198 |
| LTR84a\|Eutheria | 4.611 | 1.182 to 8.040 | **** | <0.0001 |
| LTR84b\|Eutheria | 4.258 | 0.8297 to 7.687 | *** | 0.0005 |
| LTR87\|Eutheria | 3.606 | 0.1774 to 7.035 | * | 0.0204 |
| LTR91\|Eutheria | -3.542 | -6.970 to -0.1130 | * | 0.0285 |
| LTRIS2\|Mus_mouse_genus | 3.75 | 0.3217 to 7.179 | ** | 0.0095 |
| LTRIS3\|Mus_mouse_genus | 3.829 | 0.4003 to 7.258 | ** | 0.0062 |
| LTRIS4\|Mus_mouse_genus | 4.37 | 0.9416 to 7.799 | *** | 0.0003 |
| LTRIS4A\|Mus_musculus | 3.973 | 0.5448 to 7.402 | ** | 0.0028 |
| LTRIS4B\|Mus_musculus | 4.322 | 0.8932 to 7.751 | *** | 0.0003 |
| LTRIS5\|Mus_mouse_genus | 4.472 | 1.043 to 7.901 | *** | 0.0001 |
| Lx10_3end\|Rodentia | 4.531 | 1.103 to 7.960 | **** | <0.0001 |
| Lx3_Mus_3end\|Mus_mouse_genus | 3.452 | 0.02324 to 6.881 | * | 0.0446 |
| Lx3A_3end\|Muridae | 3.448 | 0.01912 to 6.877 | * | 0.0455 |
| Lx3B_3end\|Muridae | 3.732 | 0.3031 to 7.160 | * | 0.0105 |
| Lx3C_3end\|Muridae | 3.619 | 0.1906 to 7.048 | * | 0.0191 |
| Lx4A_3end\|Muridae | 3.599 | 0.1699 to 7.027 | * | 0.0213 |
| Lx4B_3end\|Muridae | 3.995 | 0.5662 to 7.424 | ** | 0.0024 |
| Lx5_3end\|Muridae | 3.563 | 0.1338 to 6.991 | * | 0.0256 |
| Lx5b_3end\|Muridae | 3.44 | 0.01153 to 6.869 | * | 0.0473 |
| Lx6_3end\|Rodentia | 5.592 | 2.164 to 9.021 | **** | <0.0001 |
| Lx7_3end\|Rodentia | 4.709 | 1.280 to 8.138 | **** | <0.0001 |
| Lx8b_3end\|Rodentia | 4.169 | 0.7407 to 7.598 | *** | 0.0009 |
| Lx9_3end\|Rodentia | 6.258 | 2.829 to 9.686 | **** | <0.0001 |
| Mam_R4\|Mammalia | 3.604 | 0.1750 to 7.032 | * | 0.0207 |
| MER102b\|Eutheria | 3.787 | 0.3586 to 7.216 | ** | 0.0078 |
| MER102c\|Eutheria | 3.787 | 0.3586 to 7.216 | ** | 0.0078 |
| MER45A\|Eutheria | -3.54 | -6.969 to -0.1117 | * | 0.0287 |
| MER45B\|Eutheria | 4.094 | 0.6658 to 7.523 | ** | 0.0014 |
| MER47B\|Eutheria | -4.355 | -7.784 to -0.9264 | *** | 0.0003 |
| MER49\|Eutheria | 4.587 | 1.158 to 8.015 | **** | <0.0001 |
| MER50B\|Eutheria | 3.72 | 0.2912 to 7.149 | * | 0.0112 |
| MER54B\|Eutheria | 5.659 | 2.230 to 9.088 | **** | <0.0001 |
| MER57E3\|Eutheria | 5.294 | 1.866 to 8.723 | **** | <0.0001 |
| MER68\|Eutheria | 4.579 | 1.151 to 8.008 | **** | <0.0001 |
| MER70C\|Eutheria | 3.59 | 0.1617 to 7.019 | * | 0.0222 |
| MER74A\|Eutheria | 3.445 | 0.01650 to 6.874 | * | 0.0461 |
| MER92-int\|Eutheria | 5.357 | 1.928 to 8.785 | **** | <0.0001 |
| MER99\|Eutheria | 4.851 | 1.423 to 8.280 | **** | <0.0001 |
| MERV1_I\|Mus_musculus | 4.717 | 1.289 to 8.146 | **** | <0.0001 |
| MERV1_LTR\|Mus_musculus | 3.543 | 0.1139 to 6.971 | * | 0.0283 |
| MERVL_2A\|Rodentia | 3.554 | 0.1250 to 6.982 | * | 0.0268 |
| MERVL\|Mus_mouse_genus | 3.801 | 0.3726 to 7.230 | ** | 0.0072 |
| MERX\|Eutheria | 3.987 | 0.5581 to 7.416 | ** | 0.0025 |
| MIR3\|Mammalia | 3.827 | 0.3981 to 7.256 | ** | 0.0063 |
| MIRc\|Mammalia | 3.525 | 0.09632 to 6.954 | * | 0.031 |
| MLT1A0\|Eutheria | 4.117 | 0.6883 to 7.546 | ** | 0.0012 |
| MLT1A1\|Eutheria | 5.693 | 2.264 to 9.122 | **** | <0.0001 |
| MLT1H2\|Eutheria | 4.031 | 0.6020 to 7.459 | ** | 0.002 |
| MLTR13\|Murinae | 3.506 | 0.07770 to 6.935 | * | 0.034 |
| MLTR25C\|Mus_musculus | 3.697 | 0.2682 to 7.126 | * | 0.0127 |
| MLTR31A_MM\|Mus_musculus | 3.517 | 0.08807 to 6.945 | * | 0.0323 |
| MLTR31D_MM\|Mus_musculus | 4.528 | 1.100 to 7.957 | **** | <0.0001 |
| MLTR31F_MM\|Mus_musculus | 3.675 | 0.2460 to 7.103 | * | 0.0143 |
| MLTR31FA_MM\|Mus_musculus | 4.247 | 0.8185 to 7.676 | *** | 0.0005 |
| MLTR32C_MM\|Mus_musculus | 7.585 | 4.157 to 11.01 | **** | <0.0001 |
| MMERGLN_LTR\|Mus_mouse_genus | 4.751 | 1.322 to 8.180 | **** | <0.0001 |
| MMERGLN-int\|Mus_mouse_genus | 4.92 | 1.491 to 8.349 | **** | <0.0001 |
| MMERVK10C\|Mus_mouse_genus | 3.491 | 0.06275 to 6.920 | * | 0.0367 |
| MMTV-int\|Mus_mouse_genus | 8.037 | 4.608 to 11.47 | **** | <0.0001 |
| MMVL30-int\|Mus_mouse_genus | 6.614 | 3.185 to 10.04 | **** | <0.0001 |
| MRLTR33\|Murinae | 4.618 | 1.190 to 8.047 | **** | <0.0001 |
| MT2_Mm\|Mus_mouse_genus | 3.839 | 0.4104 to 7.268 | ** | 0.0059 |
| MT2A\|Rodentia | 4.696 | 1.267 to 8.124 | **** | <0.0001 |
| MT2B\|Muridae | 3.609 | 0.1800 to 7.037 | * | 0.0202 |
| MT2B1\|Muridae | 5.142 | 1.713 to 8.571 | **** | <0.0001 |
| MT2B2\|Muridae | 3.626 | 0.1973 to 7.055 | * | 0.0184 |
| MT2C_Mm\|Mus_mouse_genus | 3.679 | 0.2502 to 7.108 | * | 0.014 |
| MTC-int\|Muridae | 3.857 | 0.4280 to 7.285 | ** | 0.0053 |
| MTE2b\|Rodentia | 4.626 | 1.197 to 8.055 | **** | <0.0001 |
| MTE-int\|Rodentia | 3.989 | 0.5608 to 7.418 | ** | 0.0025 |
| MT-int\|Mus_mouse_genus | 3.432 | 0.002807 to 6.860 | * | 0.0493 |
| MuRRS4-int\|Mus_mouse_genus | 3.886 | 0.4578 to 7.315 | ** | 0.0045 |
| MusHAL1_5end\|Mus_mouse_genus | 3.614 | 0.1857 to 7.043 | * | 0.0196 |
| MusHAL1\|Mus_mouse_genus | 3.52 | 0.09095 to 6.948 | * | 0.0318 |
| MYSERV16_I\|Muridae | 3.55 | 0.1218 to 6.979 | * | 0.0272 |
| MYSERV6\|Muridae | 3.554 | 0.1249 to 6.982 | * | 0.0268 |
| ORR1A0\|Mus_mouse_genus | 3.46 | 0.03110 to 6.889 | * | 0.0429 |
| ORR1A1\|Mus_mouse_genus | 3.705 | 0.2760 to 7.133 | * | 0.0122 |
| ORR1A2\|Muridae | 3.558 | 0.1296 to 6.987 | * | 0.0262 |
| ORR1A3\|Muridae | 3.471 | 0.04271 to 6.900 | * | 0.0405 |
| ORR1A3-int\|Muridae | 3.548 | 0.1194 to 6.977 | * | 0.0275 |
| ORR1A4\|Muridae | 3.594 | 0.1654 to 7.023 | * | 0.0218 |
| ORR1B1-int\|Muridae | 3.433 | 0.004004 to 6.861 | * | 0.049 |
| ORR1B2\|Muridae | 3.442 | 0.01343 to 6.871 | * | 0.0468 |
| ORR1C1\|Muridae | 3.689 | 0.2608 to 7.118 | * | 0.0132 |
| ORR1C2\|Muridae | 3.632 | 0.2036 to 7.061 | * | 0.0178 |
| ORR1D1\|Rodentia | 3.792 | 0.3628 to 7.220 | ** | 0.0076 |
| ORR1D-int\|Rodentia | 3.535 | 0.1065 to 6.964 | * | 0.0294 |
| PB1D9\|Rodentia | 3.675 | 0.2466 to 7.104 | * | 0.0142 |
| RLTR10\|Mus_mouse_genus | 4.033 | 0.6038 to 7.461 | ** | 0.002 |
| RLTR10A\|Mus_mouse_genus | 3.661 | 0.2323 to 7.090 | * | 0.0153 |
| RLTR10C\|Mus_mouse_genus | 3.466 | 0.03711 to 6.895 | * | 0.0417 |
| RLTR10D2\|Murinae | 4.792 | 1.363 to 8.221 | **** | <0.0001 |
| RLTR10-int\|Muridae | 3.897 | 0.4680 to 7.325 | ** | 0.0042 |
| RLTR11A\|Muridae | 3.534 | 0.1054 to 6.963 | * | 0.0296 |
| RLTR11A2\|Muridae | 4.282 | 0.8531 to 7.711 | *** | 0.0004 |
| RLTR11B\|Muridae | 3.827 | 0.3980 to 7.255 | ** | 0.0063 |
| RLTR11D\|Murinae | 4.394 | 0.9652 to 7.823 | *** | 0.0002 |
| RLTR12A\|Muridae | 4.782 | 1.353 to 8.211 | **** | <0.0001 |
| RLTR12B2\|Muridae | 5.278 | 1.850 to 8.707 | **** | <0.0001 |
| RLTR12D\|Muridae | 5.199 | 1.770 to 8.628 | **** | <0.0001 |
| RLTR12E\|Muridae | 3.914 | 0.4855 to 7.343 | ** | 0.0039 |
| RLTR12F\|Muridae | 3.989 | 0.5607 to 7.418 | ** | 0.0025 |
| RLTR12G\|Muridae | 7.999 | 4.570 to 11.43 | **** | <0.0001 |
| RLTR13A3\|Mus_mouse_genus | 3.442 | 0.01311 to 6.871 | * | 0.0469 |
| RLTR13B2\|Mus_mouse_genus | 3.731 | 0.3021 to 7.160 | * | 0.0106 |
| RLTR13B3\|Mus_mouse_genus | 3.497 | 0.06828 to 6.926 | * | 0.0357 |
| RLTR13D\|Murinae | 4.383 | 0.9544 to 7.812 | *** | 0.0002 |
| RLTR13D2\|Mus_mouse_genus | 3.885 | 0.4563 to 7.314 | ** | 0.0045 |
| RLTR13D3\|Mus_mouse_genus | 3.498 | 0.06926 to 6.927 | * | 0.0355 |
| RLTR13D6\|Mus_mouse_genus | 3.848 | 0.4195 to 7.277 | ** | 0.0056 |
| RLTR14\|Muridae | 3.892 | 0.4638 to 7.321 | ** | 0.0044 |
| RLTR16\|Muridae | 4.076 | 0.6469 to 7.504 | ** | 0.0015 |
| RLTR17B_Mm\|Mus_musculus | 4.207 | 0.7785 to 7.636 | *** | 0.0007 |
| RLTR17D_Mm\|Mus_musculus | 5.741 | 2.312 to 9.170 | **** | <0.0001 |
| RLTR18-int\|Muridae | 3.737 | 0.3086 to 7.166 | * | 0.0102 |
| RLTR19A\|Muridae | 4.02 | 0.5915 to 7.449 | ** | 0.0021 |
| RLTR19B\|Muridae | 3.481 | 0.05236 to 6.910 | * | 0.0386 |
| RLTR19C\|Muridae | 5.599 | 2.171 to 9.028 | **** | <0.0001 |
| RLTR1A2_MM\|Mus_musculus | 3.832 | 0.4032 to 7.261 | ** | 0.0061 |
| RLTR1B\|Mus_mouse_genus | 3.822 | 0.3932 to 7.251 | ** | 0.0064 |
| RLTR1B-int\|Mus_mouse_genus | 4.466 | 1.038 to 7.895 | *** | 0.0001 |
| RLTR1C\|Mus_mouse_genus | 3.75 | 0.3216 to 7.179 | ** | 0.0095 |
| RLTR1D\|Mus_mouse_genus | 3.55 | 0.1215 to 6.979 | * | 0.0272 |
| RLTR1D2_MM\|Mus_musculus | 3.595 | 0.1668 to 7.024 | * | 0.0216 |
| RLTR1E_MM\|Mus_musculus | 4.219 | 0.7906 to 7.648 | *** | 0.0006 |
| RLTR1F_Mm\|Mus_musculus | 4.396 | 0.9672 to 7.825 | *** | 0.0002 |
| RLTR20A\|Muridae | 4.105 | 0.6758 to 7.533 | ** | 0.0013 |
| RLTR20A1\|Muridae | 3.876 | 0.4472 to 7.305 | ** | 0.0048 |
| RLTR20A2\|Muridae | 3.879 | 0.4506 to 7.308 | ** | 0.0047 |
| RLTR20A3_MM\|Mus_musculus | 4.232 | 0.8036 to 7.661 | *** | 0.0006 |
| RLTR20A4\|Murinae | 3.525 | 0.09676 to 6.954 | * | 0.0309 |
| RLTR20B2\|Muridae | 5.819 | 2.390 to 9.248 | **** | <0.0001 |
| RLTR20B3\|Muridae | 3.431 | 0.002025 to 6.859 | * | 0.0495 |
| RLTR20B4_MM\|Mus_musculus | 4.551 | 1.122 to 7.980 | **** | <0.0001 |
| RLTR20C\|Muridae | 5.706 | 2.277 to 9.135 | **** | <0.0001 |
| RLTR20C1_MM\|Mus_musculus | 3.542 | 0.1131 to 6.971 | * | 0.0284 |
| RLTR20D\|Muridae | 6.807 | 3.378 to 10.24 | **** | <0.0001 |
| RLTR21\|Muridae | 5.16 | 1.732 to 8.589 | **** | <0.0001 |
| RLTR23\|Muridae | 5.636 | 2.208 to 9.065 | **** | <0.0001 |
| RLTR24\|Muridae | 3.598 | 0.1694 to 7.027 | * | 0.0213 |
| RLTR24B_MM\|Mus_musculus | 5.033 | 1.604 to 8.462 | **** | <0.0001 |
| RLTR25A\|Muridae | 3.973 | 0.5445 to 7.402 | ** | 0.0028 |
| RLTR25B\|Muridae | 4.458 | 1.029 to 7.887 | *** | 0.0001 |
| RLTR26\|Muridae | 4.772 | 1.343 to 8.201 | **** | <0.0001 |
| RLTR26B_MM\|Mus_musculus | 6.733 | 3.304 to 10.16 | **** | <0.0001 |
| RLTR26C_MM\|Mus_musculus | 4.093 | 0.6638 to 7.521 | ** | 0.0014 |
| RLTR28\|Muridae | 4.066 | 0.6374 to 7.495 | ** | 0.0016 |
| RLTR3_Mm\|Mus_mouse_genus | 8.734 | 5.306 to 12.16 | **** | <0.0001 |
| RLTR30B_MM\|Mus_musculus | 3.626 | 0.1977 to 7.055 | * | 0.0184 |
| RLTR30D_MM\|Mus_musculus | 4.788 | 1.359 to 8.217 | **** | <0.0001 |
| RLTR30D2_MM\|Mus_musculus | 5.752 | 2.323 to 9.181 | **** | <0.0001 |
| RLTR31_Mm\|Mus_mouse_genus | 3.597 | 0.1682 to 7.026 | * | 0.0214 |
| RLTR31_Mur\|Muridae | 3.811 | 0.3828 to 7.240 | ** | 0.0068 |
| RLTR31A_Mm\|Mus_mouse_genus | 3.431 | 0.002496 to 6.860 | * | 0.0494 |
| RLTR31B_Mm\|Mus_mouse_genus | 4.207 | 0.7779 to 7.635 | *** | 0.0007 |
| RLTR31C_MM\|Mus_musculus | 5.402 | 1.973 to 8.830 | **** | <0.0001 |
| RLTR31M\|Murinae | 4.058 | 0.6291 to 7.486 | ** | 0.0017 |
| RLTR33\|Muridae | 3.568 | 0.1394 to 6.997 | * | 0.0249 |
| RLTR34C_MM\|Mus_musculus | 3.998 | 0.5688 to 7.426 | ** | 0.0024 |
| RLTR35B_MM\|Mus_musculus | 3.858 | 0.4293 to 7.287 | ** | 0.0053 |
| RLTR40\|Muridae | 3.632 | 0.2037 to 7.061 | * | 0.0178 |
| RLTR41\|Mus_mouse_genus | 4.287 | 0.8579 to 7.715 | *** | 0.0004 |
| RLTR41A2\|Mus_musculus | 3.921 | 0.4921 to 7.350 | ** | 0.0037 |
| RLTR41B\|Mus_musculus | 3.72 | 0.2910 to 7.148 | * | 0.0112 |
| RLTR43B\|Muridae | 4.661 | 1.232 to 8.089 | **** | <0.0001 |
| RLTR44A\|Mus_mouse_genus | 3.974 | 0.5448 to 7.402 | ** | 0.0027 |
| RLTR44B\|Mus_mouse_genus | 3.722 | 0.2935 to 7.151 | * | 0.0111 |
| RLTR45\|Mus_mouse_genus | 4.396 | 0.9675 to 7.825 | *** | 0.0002 |
| RLTR46B\|Mus_musculus | 5.207 | 1.779 to 8.636 | **** | <0.0001 |
| RLTR47_MM\|Mus_mouse_genus | 5.248 | 1.819 to 8.676 | **** | <0.0001 |
| RLTR48C\|Mus_musculus | 4.679 | 1.250 to 8.107 | **** | <0.0001 |
| RLTR49\|Mus_musculus | 4.012 | 0.5835 to 7.441 | ** | 0.0022 |
| RLTR5_Mm\|Mus_mouse_genus | 5.859 | 2.430 to 9.288 | **** | <0.0001 |
| RLTR51A_Mm\|Mus_musculus | 3.542 | 0.1130 to 6.970 | * | 0.0285 |
| RLTR51B_Mm\|Mus_musculus | 4.081 | 0.6518 to 7.509 | ** | 0.0015 |
| RLTR53_Mm\|Mus_musculus | 3.59 | 0.1610 to 7.018 | * | 0.0223 |
| RLTR6_Mm\|Mus_mouse_genus | 6.446 | 3.017 to 9.874 | **** | <0.0001 |
| RLTR6B_Mm\|Mus_musculus | 4.427 | 0.9987 to 7.856 | *** | 0.0002 |
| RLTR6C_Mm\|Mus_musculus | 5.533 | 2.104 to 8.961 | **** | <0.0001 |
| RLTR6-int\|Mus_mouse_genus | 4.982 | 1.554 to 8.411 | **** | <0.0001 |
| RLTR8\|Mus_mouse_genus | 3.541 | 0.1126 to 6.970 | * | 0.0285 |
| RLTR9A\|Mus_mouse_genus | 4 | 0.5718 to 7.429 | ** | 0.0024 |
| RLTR9B\|Mus_mouse_genus | 4.389 | 0.9608 to 7.818 | *** | 0.0002 |
| RLTR9B2\|Mus_mouse_genus | 4.207 | 0.7780 to 7.635 | *** | 0.0007 |
| RLTR9D\|Mus_mouse_genus | 3.754 | 0.3257 to 7.183 | ** | 0.0093 |
| RLTR9D2\|Mus_musculus | 4.124 | 0.6953 to 7.553 | ** | 0.0011 |
| RLTR9F\|Mus_mouse_genus | 5.955 | 2.526 to 9.384 | **** | <0.0001 |
| RLTRETN_Mm\|Mus_mouse_genus | 3.641 | 0.2124 to 7.070 | * | 0.017 |
| RMER10B\|Muridae | 4.145 | 0.7162 to 7.574 | ** | 0.001 |
| RMER12\|Muridae | 4.731 | 1.302 to 8.160 | **** | <0.0001 |
| RMER12B\|Muridae | 5.729 | 2.300 to 9.157 | **** | <0.0001 |
| RMER13A\|Muridae | 6.767 | 3.338 to 10.20 | **** | <0.0001 |
| RMER13A2\|Mus_musculus | 3.742 | 0.3134 to 7.171 | ** | 0.01 |
| RMER13B\|Muridae | 3.834 | 0.4050 to 7.262 | ** | 0.006 |
| RMER15\|Muridae | 4.04 | 0.6116 to 7.469 | ** | 0.0019 |
| RMER16_Mm\|Muridae | 3.697 | 0.2678 to 7.125 | * | 0.0127 |
| RMER16\|Muridae | 3.774 | 0.3449 to 7.202 | ** | 0.0084 |
| RMER16A2\|Muridae | 4.592 | 1.163 to 8.021 | **** | <0.0001 |
| RMER16B2\|Muridae | 5.072 | 1.643 to 8.501 | **** | <0.0001 |
| RMER17A2\|Mus_mouse_genus | 3.676 | 0.2471 to 7.105 | * | 0.0142 |
| RMER17A-int\|Mus_mouse_genus | 5.539 | 2.110 to 8.967 | **** | <0.0001 |
| RMER17B\|Muridae | 4.232 | 0.8030 to 7.660 | *** | 0.0006 |
| RMER17B2\|Mus_musculus | 3.771 | 0.3426 to 7.200 | ** | 0.0085 |
| RMER17C\|Muridae | 3.949 | 0.5206 to 7.378 | ** | 0.0032 |
| RMER17C2\|Muridae | 3.53 | 0.1014 to 6.959 | * | 0.0302 |
| RMER17C-int\|Muridae | 4.248 | 0.8196 to 7.677 | *** | 0.0005 |
| RMER17D\|Muridae | 3.683 | 0.2543 to 7.112 | * | 0.0137 |
| RMER17D2\|Muridae | 3.882 | 0.4533 to 7.311 | ** | 0.0046 |
| RMER19A\|Muridae | 3.631 | 0.2025 to 7.060 | * | 0.0179 |
| RMER19B\|Muridae | 3.768 | 0.3389 to 7.196 | ** | 0.0087 |
| RMER19B2\|Muridae | -3.627 | -7.056 to -0.1987 | * | 0.0183 |
| RMER19C\|Muridae | 3.8 | 0.3712 to 7.229 | ** | 0.0073 |
| RMER1A\|Muridae | 3.595 | 0.1662 to 7.024 | * | 0.0217 |
| RMER1B\|Muridae | 3.71 | 0.2810 to 7.138 | * | 0.0118 |
| RMER1C\|Muridae | 8.625 | 5.197 to 12.05 | **** | <0.0001 |
| RMER20C_Mm\|Muridae | 3.583 | 0.1542 to 7.012 | * | 0.0231 |
| RMER21A\|Muridae | -3.698 | -7.127 to -0.2698 | * | 0.0126 |
| RMER3D4\|Muridae | -3.574 | -7.002 to -0.1450 | * | 0.0242 |
| RMER4A\|Muridae | 3.731 | 0.3024 to 7.160 | * | 0.0106 |
| RMER5\|Rodentia | 5.97 | 2.542 to 9.399 | **** | <0.0001 |
| RMER6A\|Muridae | 3.592 | 0.1629 to 7.020 | * | 0.022 |
| RMER6B\|Muridae | 4.102 | 0.6737 to 7.531 | ** | 0.0013 |
| RMER6BA\|Murinae | 4.818 | 1.390 to 8.247 | **** | <0.0001 |
| RMER6C\|Muridae | 3.626 | 0.1969 to 7.054 | * | 0.0185 |
| RodERV21\|Rodentia | 5.179 | 1.750 to 8.607 | **** | <0.0001 |
| Row 251 | 4.72 | 1.291 to 8.148 | **** | <0.0001 |
| RSINE1\|Muridae | 4.288 | 0.8596 to 7.717 | *** | 0.0004 |
| SRV_MM-int\|Mus_mouse_genus | 5.144 | 1.715 to 8.572 | **** | <0.0001 |
| Tigger10\|Mammalia | 4.129 | 0.7000 to 7.557 | ** | 0.0011 |
| Tigger18a\|Mammalia | 4.445 | 1.016 to 7.874 | *** | 0.0002 |
| Tigger6a\|Eutheria | 4.557 | 1.129 to 7.986 | **** | <0.0001 |
| Tigger7\|Eutheria | 4.131 | 0.7026 to 7.560 | ** | 0.0011 |
| Zaphod\|Eutheria | 3.495 | 0.06657 to 6.924 | * | 0.036 |
| Zaphod3\|Eutheria | 3.62 | 0.1917 to 7.049 | * | 0.019 |
| ZP3AR\|Muridae | 3.985 | 0.5563 to 7.414 | ** | 0.0026 |

^†^p-values adjusted using Sidak’s multiple comparisons test
