# Supplementary material for: 5-Azacytidine Potentiates Anti-tumor Immunity in a Model of Pancreatic Ductal Adenocarcinoma
Source: Front Immunol. 2020 Mar 31;11:538. doi: 10.3389/fimmu.2020.00538 (PMC7136411; doi:10.3389/fimmu.2020.00538)
Supplement: Supplementary file 2 [file Table_2.DOCX]

**Supplemental Table 2.** Mean differences in fold change in expression of transposable elements during transition from acinar-to-ductal metaplasia (ADM) to pancreatic ductal adenocarcinoma (PDAC).

| **Unique TE ID** | **Fold Change** | **95.00% CI of diff.** | **Summary** | **Adjusted P Value^†^** |
| --- | --- | --- | --- | --- |
| **PDAC/ADM** | | | | |
| 4.5SRNA\|Rodentia | -12.38 | -16.81 to -7.944 | **** | <0.0001 |
| 7SK\|Eutheria | -13.55 | -17.98 to -9.115 | **** | <0.0001 |
| 7SLRNA_short_\|Rodentia | -14.98 | -19.41 to -10.55 | **** | <0.0001 |
| 7SLRNA\|Eutheria | -20.22 | -24.65 to -15.78 | **** | <0.0001 |
| Arthur1C\|Eutheria | 11.8 | 7.370 to 16.24 | **** | <0.0001 |
| B1_Mm\|Mus_mouse_genus | -10.38 | -14.82 to -5.951 | **** | <0.0001 |
| B1_Mur2\|Muridae | -8.903 | -13.34 to -4.470 | **** | <0.0001 |
| B1_Mur4\|Muridae | -14.9 | -19.33 to -10.46 | **** | <0.0001 |
| B1_Mus1\|Mus_mouse_genus | -9.189 | -13.62 to -4.756 | **** | <0.0001 |
| B1_Mus2\|Mus_mouse_genus | -4.978 | -9.412 to -0.5445 | ** | 0.0055 |
| B1F\|Muridae | -5.587 | -10.02 to -1.153 | *** | 0.0003 |
| B1F1\|Muridae | -5.311 | -9.744 to -0.8770 | ** | 0.0013 |
| B2_Mm1a\|Mus_mouse_genus | -6.307 | -10.74 to -1.874 | **** | <0.0001 |
| B2_Mm1t\|Mus_mouse_genus | -6.905 | -11.34 to -2.471 | **** | <0.0001 |
| B2_Mm2\|Mus_mouse_genus | -9.251 | -13.68 to -4.817 | **** | <0.0001 |
| B3A\|Muridae | -5.365 | -9.799 to -0.9317 | *** | 0.001 |
| BC1_Mm\|Mus_mouse_genus | -9.867 | -14.30 to -5.433 | **** | <0.0001 |
| BGLII_B\|Muridae | -5.033 | -9.467 to -0.5995 | ** | 0.0044 |
| BGLII_C\|Mus_musculus | -4.831 | -9.264 to -0.3969 | * | 0.0103 |
| BGLII_Mur\|Murinae | 6.7 | 2.266 to 11.13 | **** | <0.0001 |
| BGLII_Mus\|Mus_mouse_genus | -8.893 | -13.33 to -4.460 | **** | <0.0001 |
| BGLII\|Mus_mouse_genus | -5.019 | -9.452 to -0.5850 | ** | 0.0046 |
| C573_MM\|Mus_mouse_genus | 11.24 | 6.809 to 15.68 | **** | <0.0001 |
| Charlie10b\|Eutheria | 7.437 | 3.003 to 11.87 | **** | <0.0001 |
| Charlie11\|Eutheria | -4.522 | -8.955 to -0.08821 | * | 0.0357 |
| Charlie15a\|Mammalia | 7.741 | 3.307 to 12.17 | **** | <0.0001 |
| Charlie15b\|Mammalia | 7.213 | 2.780 to 11.65 | **** | <0.0001 |
| Charlie18a\|Mammalia | 7.126 | 2.692 to 11.56 | **** | <0.0001 |
| Charlie19a\|Mammalia | 17.4 | 12.97 to 21.84 | **** | <0.0001 |
| Charlie1a\|Eutheria | 12.32 | 7.885 to 16.75 | **** | <0.0001 |
| Charlie2a\|Eutheria | 8.091 | 3.657 to 12.52 | **** | <0.0001 |
| Charlie5\|Eutheria | 6.298 | 1.865 to 10.73 | **** | <0.0001 |
| Charlie7a\|Eutheria | 8.867 | 4.434 to 13.30 | **** | <0.0001 |
| Charlie9\|Eutheria | 8.67 | 4.237 to 13.10 | **** | <0.0001 |
| CYRA11_Mm\|Mus_mouse_genus | 5.14 | 0.7063 to 9.573 | ** | 0.0027 |
| DNA1_Mam\|Eutheria | 14.06 | 9.625 to 18.49 | **** | <0.0001 |
| ERV3-16A3_LTR\|Eutheria | 11.72 | 7.285 to 16.15 | **** | <0.0001 |
| ERVB2_1A-I_MM\|Mus_musculus | -4.7 | -9.133 to -0.2663 | * | 0.0176 |
| ERVB4_1B-I_MM\|Mus_musculus | -8.566 | -13.00 to -4.133 | **** | <0.0001 |
| ERVB4_1B-LTR_MM\|Mus_musculus | -7.828 | -12.26 to -3.394 | **** | <0.0001 |
| ERVB4_3-I_MM\|Mus_musculus | -5.365 | -9.799 to -0.9314 | *** | 0.001 |
| ERVB5_1-I_MM\|Mus_mouse_genus | -6.574 | -11.01 to -2.141 | **** | <0.0001 |
| ERVB5_2-LTR_MM\|Mus_musculus | -9.908 | -14.34 to -5.474 | **** | <0.0001 |
| ERVB7_1-LTR_MM\|Mus_musculus | -10.66 | -15.09 to -6.224 | **** | <0.0001 |
| ERVB7_2-LTR_MM\|Mus_musculus | -5.585 | -10.02 to -1.151 | *** | 0.0004 |
| ERVB7_3-LTR_MM\|Mus_musculus | -8.846 | -13.28 to -4.412 | **** | <0.0001 |
| ERVL\|Eutheria | 4.776 | 0.3420 to 9.209 | * | 0.0129 |
| ERVL-B4\|Eutheria | 8.227 | 3.793 to 12.66 | **** | <0.0001 |
| ETnERV\|Mus_musculus | -13.26 | -17.70 to -8.829 | **** | <0.0001 |
| ETnERV3\|Mus_mouse_genus | -6.783 | -11.22 to -2.350 | **** | <0.0001 |
| FordPrefect_a\|Eutheria | 8.037 | 3.603 to 12.47 | **** | <0.0001 |
| FordPrefect\|Eutheria | 5.471 | 1.038 to 9.905 | *** | 0.0006 |
| HAL1ME\|Mammalia | 5.947 | 1.513 to 10.38 | **** | <0.0001 |
| hAT-N1_Mam\|Mammalia | 12.64 | 8.207 to 17.07 | **** | <0.0001 |
| HERVL32\|Eutheria | 6.892 | 2.458 to 11.33 | **** | <0.0001 |
| IAP1-MM_I\|Mus_musculus | -5.391 | -9.824 to -0.9571 | *** | 0.0009 |
| IAP1-MM_LTR\|Mus_musculus | -6.782 | -11.22 to -2.349 | **** | <0.0001 |
| IAPEY3-int\|Mus_mouse_genus | -11.14 | -15.57 to -6.707 | **** | <0.0001 |
| IAPEY4_I\|Mus_musculus | -7.862 | -12.30 to -3.429 | **** | <0.0001 |
| IAPEz-int\|Mus_mouse_genus | -6.968 | -11.40 to -2.535 | **** | <0.0001 |
| IAPLTR1a_Mm\|Mus_mouse_genus | -9.808 | -14.24 to -5.374 | **** | <0.0001 |
| IAPLTR2_Mm\|Mus_mouse_genus | -8.385 | -12.82 to -3.952 | **** | <0.0001 |
| IAPLTR2a\|Mus_mouse_genus | -8.461 | -12.89 to -4.028 | **** | <0.0001 |
| IAPLTR3-int\|Mus_mouse_genus | 7.093 | 2.659 to 11.53 | **** | <0.0001 |
| IAPLTR4\|Mus_mouse_genus | -4.861 | -9.294 to -0.4270 | ** | 0.0091 |
| IMPB_01\|Mus_musculus | -10.32 | -14.75 to -5.883 | **** | <0.0001 |
| Kanga2_a\|Eutheria | 5.386 | 0.9522 to 9.819 | *** | 0.0009 |
| L1_Mm_orf2\|Mus_mouse_genus | -9.268 | -13.70 to -4.835 | **** | <0.0001 |
| L1_Mur1_orf2\|Muridae | -7.204 | -11.64 to -2.771 | **** | <0.0001 |
| L1_Mur2_orf2\|Muridae | -12.28 | -16.71 to -7.844 | **** | <0.0001 |
| L1_Mur3_orf2\|Muridae | 4.824 | 0.3906 to 9.258 | * | 0.0106 |
| L1_Mus1_3end\|Mus_mouse_genus | -5.13 | -9.563 to -0.6960 | ** | 0.0029 |
| L1_Mus2_3end\|Mus_mouse_genus | -5.178 | -9.611 to -0.7443 | ** | 0.0023 |
| L1_Mus3_3end\|Mus_mouse_genus | -5.79 | -10.22 to -1.356 | *** | 0.0001 |
| L1_Mus3_5end\|Mus_mouse_genus | -9.829 | -14.26 to -5.395 | **** | <0.0001 |
| L1_Mus3_orf2\|Mus_mouse_genus | -10.83 | -15.27 to -6.401 | **** | <0.0001 |
| L1_Mus4_3end\|Mus_mouse_genus | -8.302 | -12.74 to -3.869 | **** | <0.0001 |
| L1M2_orf2\|Eutheria | 15.2 | 10.77 to 19.63 | **** | <0.0001 |
| L1M3b_5end\|Eutheria | 5.86 | 1.427 to 10.29 | **** | <0.0001 |
| L1M3e_5end\|Eutheria | 8.959 | 4.525 to 13.39 | **** | <0.0001 |
| L1M4_5end\|Eutheria | -5.096 | -9.529 to -0.6621 | ** | 0.0033 |
| L1M4a2_5end\|Eutheria | 8.614 | 4.180 to 13.05 | **** | <0.0001 |
| L1M4b_5end\|Eutheria | 7.312 | 2.878 to 11.75 | **** | <0.0001 |
| L1M5_orf2\|Eutheria | 5.069 | 0.6354 to 9.503 | ** | 0.0037 |
| L1M6_5end\|Mammalia | 6.336 | 1.902 to 10.77 | **** | <0.0001 |
| L1M6B_5end\|Mammalia | -4.644 | -9.077 to -0.2100 | * | 0.0221 |
| L1M7_5end\|Mammalia | 8.594 | 4.160 to 13.03 | **** | <0.0001 |
| L1MA4_3end\|Eutheria | 6.248 | 1.814 to 10.68 | **** | <0.0001 |
| L1MA4A_3end\|Eutheria | 6.26 | 1.826 to 10.69 | **** | <0.0001 |
| L1MA5A_3end\|Eutheria | 5.089 | 0.6554 to 9.523 | ** | 0.0034 |
| L1MA7_3end\|Eutheria | 8.318 | 3.885 to 12.75 | **** | <0.0001 |
| L1MB4_3end\|Eutheria | 18.22 | 13.79 to 22.66 | **** | <0.0001 |
| L1MB8_3end\|Eutheria | 4.963 | 0.5296 to 9.397 | ** | 0.0059 |
| L1MC3_3end\|Eutheria | 5.475 | 1.042 to 9.909 | *** | 0.0006 |
| L1MC5_3end\|Eutheria | 6.716 | 2.283 to 11.15 | **** | <0.0001 |
| L1MCb_5end\|Eutheria | 14.39 | 9.953 to 18.82 | **** | <0.0001 |
| L1Md_A_5end\|Mus_mouse_genus | -13.77 | -18.20 to -9.333 | **** | <0.0001 |
| L1Md_F_5end\|Mus_mouse_genus | -4.572 | -9.006 to -0.1386 | * | 0.0293 |
| L1Md_F2_3end\|Mus_mouse_genus | -7.587 | -12.02 to -3.153 | **** | <0.0001 |
| L1Md_F3_3end\|Mus_mouse_genus | -14.51 | -18.95 to -10.08 | **** | <0.0001 |
| L1Md_Gf_5end\|Mus_mouse_genus | -4.861 | -9.294 to -0.4271 | ** | 0.0091 |
| L1Md_T_3end\|Mus_mouse_genus | -5.774 | -10.21 to -1.341 | *** | 0.0001 |
| L1MDa_5end\|Eutheria | 7.155 | 2.721 to 11.59 | **** | <0.0001 |
| L1ME1_3end\|Eutheria | 8.506 | 4.072 to 12.94 | **** | <0.0001 |
| L1ME2_3end\|Eutheria | 5.381 | 0.9474 to 9.815 | *** | 0.0009 |
| L1ME3_3end\|Eutheria | 5.644 | 1.210 to 10.08 | *** | 0.0003 |
| L1ME3C_3end\|Eutheria | 11.18 | 6.744 to 15.61 | **** | <0.0001 |
| L1ME3D_3end\|Eutheria | 8.7 | 4.267 to 13.13 | **** | <0.0001 |
| L1ME4a_3end\|Eutheria | 7.357 | 2.923 to 11.79 | **** | <0.0001 |
| L1ME4c_3end\|Eutheria | 18.41 | 13.98 to 22.84 | **** | <0.0001 |
| L1MEa_5end\|Eutheria | 7.737 | 3.303 to 12.17 | **** | <0.0001 |
| L1MEg_5end\|Mammalia | 8.125 | 3.691 to 12.56 | **** | <0.0001 |
| L1MEh_5end\|Mammalia | 7.781 | 3.347 to 12.21 | **** | <0.0001 |
| L1VL1_5end\|Mus_mouse_genus | -5.026 | -9.460 to -0.5923 | ** | 0.0045 |
| L1VL4_5end\|Muridae | -7.488 | -11.92 to -3.054 | **** | <0.0001 |
| L2a_3end\|Mammalia | 10.21 | 5.773 to 14.64 | **** | <0.0001 |
| L4_A_Mam\|Mammalia | 5.615 | 1.182 to 10.05 | *** | 0.0003 |
| LSU-rRNA_Hsa\|Metazoa | -14.67 | -19.10 to -10.24 | **** | <0.0001 |
| LTR102_Mam\|Mammalia | 9.999 | 5.565 to 14.43 | **** | <0.0001 |
| LTR103b_Mam\|Mammalia | 8.59 | 4.156 to 13.02 | **** | <0.0001 |
| LTR105_Mam\|Mammalia | 7.43 | 2.997 to 11.86 | **** | <0.0001 |
| LTR16B1\|Eutheria | 14.21 | 9.779 to 18.65 | **** | <0.0001 |
| LTR16E2\|Eutheria | 13.73 | 9.298 to 18.17 | **** | <0.0001 |
| LTR28\|Eutheria | 7.23 | 2.796 to 11.66 | **** | <0.0001 |
| LTR33\|Eutheria | 14.54 | 10.11 to 18.98 | **** | <0.0001 |
| LTR33C\|Eutheria | 6.604 | 2.171 to 11.04 | **** | <0.0001 |
| LTR34\|Eutheria | 11.77 | 7.337 to 16.20 | **** | <0.0001 |
| LTR37A\|Eutheria | 4.912 | 0.4781 to 9.345 | ** | 0.0073 |
| LTR41B\|Eutheria | 9.866 | 5.432 to 14.30 | **** | <0.0001 |
| LTR44\|Eutheria | 10.17 | 5.735 to 14.60 | **** | <0.0001 |
| LTR48B\|Eutheria | 4.593 | 0.1593 to 9.026 | * | 0.027 |
| LTR52\|Eutheria | 9.771 | 5.337 to 14.20 | **** | <0.0001 |
| LTR55\|Eutheria | 4.976 | 0.5425 to 9.410 | ** | 0.0056 |
| LTR59\|Eutheria | 4.697 | 0.2636 to 9.131 | * | 0.0178 |
| LTR64\|Eutheria | 5.381 | 0.9478 to 9.815 | *** | 0.0009 |
| LTR67B\|Eutheria | -4.907 | -9.340 to -0.4732 | ** | 0.0075 |
| LTR73\|Eutheria | 4.74 | 0.3064 to 9.174 | * | 0.015 |
| LTR75_1\|Eutheria | 5.892 | 1.458 to 10.33 | **** | <0.0001 |
| LTR78\|Mammalia | 5.091 | 0.6578 to 9.525 | ** | 0.0034 |
| LTR78B\|Eutheria | 5.381 | 0.9470 to 9.814 | *** | 0.0009 |
| LTR81A\|Mammalia | 8.056 | 3.623 to 12.49 | **** | <0.0001 |
| LTR81C\|Mammalia | 9.316 | 4.882 to 13.75 | **** | <0.0001 |
| LTR9\|Eutheria | 6.938 | 2.505 to 11.37 | **** | <0.0001 |
| LTRIS_Mm\|Mus_mouse_genus | -11.39 | -15.82 to -6.955 | **** | <0.0001 |
| LTRIS_Mus\|Mus_mouse_genus | -13.36 | -17.79 to -8.928 | **** | <0.0001 |
| LTRIS2\|Mus_mouse_genus | -8.766 | -13.20 to -4.332 | **** | <0.0001 |
| LTRIS3\|Mus_mouse_genus | -6.748 | -11.18 to -2.314 | **** | <0.0001 |
| LTRIS4B\|Mus_musculus | -7.293 | -11.73 to -2.860 | **** | <0.0001 |
| LTRIS5\|Mus_mouse_genus | -8.479 | -12.91 to -4.046 | **** | <0.0001 |
| LTRIS6\|Mus_musculus | -7.587 | -12.02 to -3.154 | **** | <0.0001 |
| Lx2_3end\|Muridae | -8.301 | -12.73 to -3.867 | **** | <0.0001 |
| Lx3_Mus_3end\|Mus_mouse_genus | -5.313 | -9.746 to -0.8792 | ** | 0.0013 |
| Lx3A_3end\|Muridae | -6.125 | -10.56 to -1.692 | **** | <0.0001 |
| Lx3C_3end\|Muridae | -9.007 | -13.44 to -4.574 | **** | <0.0001 |
| Lx4A_3end\|Muridae | -4.825 | -9.258 to -0.3911 | * | 0.0106 |
| Lx4B_3end\|Muridae | -5.329 | -9.763 to -0.8956 | ** | 0.0012 |
| Lx5_3end\|Muridae | 5.223 | 0.7897 to 9.657 | ** | 0.0019 |
| Mam_R4\|Mammalia | 7.948 | 3.515 to 12.38 | **** | <0.0001 |
| MamGyp-int\|Mammalia | 5.345 | 0.9112 to 9.778 | ** | 0.0011 |
| MamGypLTR1c\|Mammalia | 15.06 | 10.62 to 19.49 | **** | <0.0001 |
| MamRep564\|Eutheria | 7.417 | 2.984 to 11.85 | **** | <0.0001 |
| MamTip1\|Mammalia | 8.445 | 4.012 to 12.88 | **** | <0.0001 |
| MER101B\|Eutheria | 8.049 | 3.615 to 12.48 | **** | <0.0001 |
| MER102a\|Eutheria | 8.552 | 4.118 to 12.99 | **** | <0.0001 |
| MER103C\|Eutheria | 5.372 | 0.9385 to 9.806 | *** | 0.001 |
| MER105\|Eutheria | 4.665 | 0.2312 to 9.098 | * | 0.0203 |
| MER106A\|Eutheria | 6.28 | 1.846 to 10.71 | **** | <0.0001 |
| MER106B\|Eutheria | 8.22 | 3.786 to 12.65 | **** | <0.0001 |
| MER110A\|Eutheria | 10.94 | 6.502 to 15.37 | **** | <0.0001 |
| MER112\|Eutheria | 5.255 | 0.2976 to 10.21 | * | 0.0176 |
| MER113B\|Eutheria | 5.044 | 0.6101 to 9.477 | ** | 0.0042 |
| MER121\|Mammalia | 11.28 | 6.845 to 15.71 | **** | <0.0001 |
| MER3\|Eutheria | 7.189 | 2.756 to 11.62 | **** | <0.0001 |
| MER33\|Eutheria | 5.219 | 0.7854 to 9.653 | ** | 0.0019 |
| MER34\|Eutheria | 7.448 | 3.014 to 11.88 | **** | <0.0001 |
| MER34A\|Eutheria | 9.759 | 5.325 to 14.19 | **** | <0.0001 |
| MER34A1\|Eutheria | 4.896 | 0.4628 to 9.330 | ** | 0.0078 |
| MER34B-int\|Eutheria | -5.189 | -9.623 to -0.7557 | ** | 0.0022 |
| MER44C\|Eutheria | 9.189 | 4.756 to 13.62 | **** | <0.0001 |
| MER45A\|Eutheria | 5.848 | 1.414 to 10.28 | **** | <0.0001 |
| MER45R\|Eutheria | 4.724 | 0.2909 to 9.158 | * | 0.0159 |
| MER47B\|Eutheria | 22.85 | 18.42 to 27.28 | **** | <0.0001 |
| MER57D\|Eutheria | 6.671 | 2.237 to 11.10 | **** | <0.0001 |
| MER58A\|Eutheria | 5.134 | 0.7007 to 9.568 | ** | 0.0028 |
| MER58B\|Eutheria | 10.99 | 6.552 to 15.42 | **** | <0.0001 |
| MER58C\|Eutheria | 6.172 | 1.739 to 10.61 | **** | <0.0001 |
| MER5A\|Eutheria_Mammalia | 5.241 | 0.8073 to 9.674 | ** | 0.0017 |
| MER5A1\|Eutheria | 9.772 | 5.339 to 14.21 | **** | <0.0001 |
| MER63B\|Eutheria | 15.88 | 11.44 to 20.31 | **** | <0.0001 |
| MER65D\|Eutheria | 4.467 | 0.03360 to 8.901 | * | 0.044 |
| MER66A\|Eutheria | 9.035 | 4.601 to 13.47 | **** | <0.0001 |
| MER66B\|Eutheria | 11.07 | 6.632 to 15.50 | **** | <0.0001 |
| MER66C\|Eutheria | 14.16 | 9.724 to 18.59 | **** | <0.0001 |
| MER67C\|Eutheria | 4.638 | 0.2042 to 9.071 | * | 0.0226 |
| MER67D\|Eutheria | 8.44 | 4.007 to 12.87 | **** | <0.0001 |
| MER73\|Eutheria | 7.267 | 2.833 to 11.70 | **** | <0.0001 |
| MER74A\|Eutheria | 11.04 | 6.607 to 15.47 | **** | <0.0001 |
| MER74B\|Eutheria | 6.056 | 1.623 to 10.49 | **** | <0.0001 |
| MER74C\|Eutheria | 6.207 | 1.773 to 10.64 | **** | <0.0001 |
| MER76\|Eutheria | 13.71 | 9.272 to 18.14 | **** | <0.0001 |
| MER91B\|Eutheria_Mammalia | 5.247 | 0.8138 to 9.681 | ** | 0.0017 |
| MER92C\|Eutheria | 8.757 | 4.323 to 13.19 | **** | <0.0001 |
| MER96\|Eutheria | 13.16 | 8.725 to 17.59 | **** | <0.0001 |
| MER97a\|Eutheria | 12.8 | 8.363 to 17.23 | **** | <0.0001 |
| MER97b\|Eutheria | 8.052 | 3.618 to 12.49 | **** | <0.0001 |
| MERV1_I\|Mus_musculus | -8.033 | -12.47 to -3.599 | **** | <0.0001 |
| MERV1_LTR\|Mus_musculus | -7.282 | -11.72 to -2.848 | **** | <0.0001 |
| MERVL_2A\|Rodentia | -10.34 | -14.77 to -5.908 | **** | <0.0001 |
| MERVL\|Mus_mouse_genus | -6.949 | -11.38 to -2.516 | **** | <0.0001 |
| MIR\|Mammalia | 5.719 | 1.285 to 10.15 | *** | 0.0002 |
| MLT1A\|Eutheria | 8.005 | 3.571 to 12.44 | **** | <0.0001 |
| MLT1E1A\|Eutheria | 7.675 | 3.242 to 12.11 | **** | <0.0001 |
| MLT1E2\|Eutheria | 17.36 | 12.93 to 21.80 | **** | <0.0001 |
| MLT1G1\|Eutheria | 5.147 | 0.7138 to 9.581 | ** | 0.0026 |
| MLT1J2\|Eutheria | 9.79 | 5.357 to 14.22 | **** | <0.0001 |
| MLT2B3\|Eutheria | 6.824 | 2.391 to 11.26 | **** | <0.0001 |
| MLT2F\|Eutheria | 14.41 | 9.981 to 18.85 | **** | <0.0001 |
| MLT-int\|Eutheria | -4.971 | -9.404 to -0.5373 | ** | 0.0057 |
| MLTR13\|Murinae | -5.463 | -9.896 to -1.029 | *** | 0.0006 |
| MLTR25A\|Mus_musculus | -5.988 | -10.42 to -1.555 | **** | <0.0001 |
| MLTR31A_MM\|Mus_musculus | 9.004 | 4.570 to 13.44 | **** | <0.0001 |
| MLTR31D_MM\|Mus_musculus | -6.808 | -11.24 to -2.374 | **** | <0.0001 |
| MLTR31E_MM\|Mus_musculus | 5.146 | 0.7129 to 9.580 | ** | 0.0026 |
| MLTR73\|Murinae | 4.845 | 0.4113 to 9.278 | ** | 0.0097 |
| MMERGLN_LTR\|Mus_mouse_genus | -11.34 | -15.77 to -6.907 | **** | <0.0001 |
| MMERGLN-int\|Mus_mouse_genus | -16.38 | -20.82 to -11.95 | **** | <0.0001 |
| MMERVK9C_I\|Mus_musculus | -7.637 | -12.07 to -3.203 | **** | <0.0001 |
| MMETn-int\|Mus_mouse_genus | -5.231 | -9.665 to -0.7977 | ** | 0.0018 |
| MMTV-int\|Mus_mouse_genus | -7.599 | -12.03 to -3.166 | **** | <0.0001 |
| MMVL30-int\|Mus_mouse_genus | -14.57 | -19.00 to -10.13 | **** | <0.0001 |
| MT2_Mm\|Mus_mouse_genus | -8.723 | -13.16 to -4.290 | **** | <0.0001 |
| MT2B\|Muridae | -7.463 | -11.90 to -3.029 | **** | <0.0001 |
| MT2C_Mm\|Mus_mouse_genus | -9.018 | -13.45 to -4.584 | **** | <0.0001 |
| MTB_Mm\|Mus_mouse_genus | -6.743 | -11.18 to -2.310 | **** | <0.0001 |
| MTB\|Muridae | -5.511 | -9.945 to -1.077 | *** | 0.0005 |
| MTC-int\|Muridae | -4.75 | -9.183 to -0.3162 | * | 0.0144 |
| MTD\|Rodentia | 8.571 | 4.138 to 13.00 | **** | <0.0001 |
| MTE2a\|Rodentia | 5.084 | 0.6506 to 9.518 | ** | 0.0035 |
| MT-int\|Mus_mouse_genus | -11.42 | -15.85 to -6.987 | **** | <0.0001 |
| MuLV-int\|Mus_mouse_genus | -16.7 | -21.14 to -12.27 | **** | <0.0001 |
| MuRRS-int\|Mus_mouse_genus | -7.072 | -11.51 to -2.638 | **** | <0.0001 |
| MYSERV\|Muridae | -5.276 | -9.710 to -0.8428 | ** | 0.0015 |
| ORR1A0\|Mus_mouse_genus | -6.002 | -10.44 to -1.568 | **** | <0.0001 |
| ORR1A1\|Mus_mouse_genus | -5.49 | -9.924 to -1.057 | *** | 0.0006 |
| ORR1A1-int\|Mus_mouse_genus | -11.53 | -15.96 to -7.096 | **** | <0.0001 |
| ORR1A2\|Muridae | -7.857 | -12.29 to -3.424 | **** | <0.0001 |
| ORR1B1\|Muridae | -5.182 | -9.616 to -0.7488 | ** | 0.0023 |
| ORR1B1-int\|Muridae | 7.176 | 2.743 to 11.61 | **** | <0.0001 |
| ORR1C2\|Muridae | -8.437 | -12.87 to -4.003 | **** | <0.0001 |
| ORR1D1\|Rodentia | -7.892 | -12.33 to -3.458 | **** | <0.0001 |
| ORR1F\|Murinae | 7.858 | 3.424 to 12.29 | **** | <0.0001 |
| ORR1G\|Murinae | 4.663 | 0.2298 to 9.097 | * | 0.0204 |
| PB1\|Rodentia | -12.11 | -16.54 to -7.673 | **** | <0.0001 |
| PB1D9\|Rodentia | -5.322 | -9.755 to -0.8880 | ** | 0.0012 |
| RLTR10A\|Mus_mouse_genus | -5.867 | -10.30 to -1.433 | **** | <0.0001 |
| RLTR10B\|Mus_mouse_genus | 4.732 | 0.2981 to 9.165 | * | 0.0155 |
| RLTR10B2\|Mus_mouse_genus | -6.765 | -11.20 to -2.331 | **** | <0.0001 |
| RLTR10C\|Mus_mouse_genus | -10.41 | -14.84 to -5.976 | **** | <0.0001 |
| RLTR10D2\|Murinae | -8.663 | -13.10 to -4.230 | **** | <0.0001 |
| RLTR10-int\|Muridae | -9.05 | -13.48 to -4.616 | **** | <0.0001 |
| RLTR11B\|Muridae | -7.72 | -12.15 to -3.286 | **** | <0.0001 |
| RLTR11C_MM\|Mus_musculus | 5.856 | 1.422 to 10.29 | **** | <0.0001 |
| RLTR12B\|Muridae | 5.387 | 0.9534 to 9.821 | *** | 0.0009 |
| RLTR12C\|Muridae | 8.834 | 4.401 to 13.27 | **** | <0.0001 |
| RLTR12F\|Muridae | 7.381 | 2.947 to 11.81 | **** | <0.0001 |
| RLTR12H\|Muridae | 9.562 | 5.128 to 14.00 | **** | <0.0001 |
| RLTR13B2\|Mus_mouse_genus | -6.267 | -10.70 to -1.833 | **** | <0.0001 |
| RLTR13B4\|Mus_mouse_genus | -9.479 | -13.91 to -5.046 | **** | <0.0001 |
| RLTR13C2\|Mus_mouse_genus | 4.585 | 0.1513 to 9.018 | * | 0.0279 |
| RLTR13D\|Murinae | -6.226 | -10.66 to -1.792 | **** | <0.0001 |
| RLTR13D2\|Mus_mouse_genus | -6.928 | -11.36 to -2.495 | **** | <0.0001 |
| RLTR13D3A\|Mus_musculus | 5.522 | 1.088 to 9.956 | *** | 0.0005 |
| RLTR13D4\|Mus_mouse_genus | -8.183 | -12.62 to -3.750 | **** | <0.0001 |
| RLTR13D6\|Mus_mouse_genus | -4.8 | -9.234 to -0.3668 | * | 0.0117 |
| RLTR14_RN\|Murinae | 6.21 | 1.777 to 10.64 | **** | <0.0001 |
| RLTR16\|Muridae | 6.232 | 1.798 to 10.67 | **** | <0.0001 |
| RLTR16C_MM\|Mus_musculus | 12.64 | 8.203 to 17.07 | **** | <0.0001 |
| RLTR17\|Muridae | -7.688 | -12.12 to -3.254 | **** | <0.0001 |
| RLTR19\|Muridae | -4.494 | -8.927 to -0.06001 | * | 0.0398 |
| RLTR19A\|Muridae | 4.572 | 0.1386 to 9.006 | * | 0.0293 |
| RLTR19B\|Muridae | 6.713 | 2.280 to 11.15 | **** | <0.0001 |
| RLTR19C\|Muridae | -5.381 | -9.815 to -0.9477 | *** | 0.0009 |
| RLTR19-int\|Muridae | -7.08 | -11.51 to -2.647 | **** | <0.0001 |
| RLTR1A2_MM\|Mus_musculus | 5.994 | 1.560 to 10.43 | **** | <0.0001 |
| RLTR1B\|Mus_mouse_genus | -5.005 | -9.438 to -0.5711 | ** | 0.0049 |
| RLTR1B-int\|Mus_mouse_genus | -8.011 | -12.44 to -3.578 | **** | <0.0001 |
| RLTR1C\|Mus_mouse_genus | -7.365 | -11.80 to -2.932 | **** | <0.0001 |
| RLTR1D\|Mus_mouse_genus | 6.611 | 2.178 to 11.04 | **** | <0.0001 |
| RLTR20B3\|Muridae | 4.859 | 0.4249 to 9.292 | ** | 0.0092 |
| RLTR20B4_MM\|Mus_musculus | -5.16 | -9.594 to -0.7266 | ** | 0.0025 |
| RLTR20B5_MM\|Mus_musculus | 7.566 | 3.133 to 12.00 | **** | <0.0001 |
| RLTR21\|Muridae | -6.859 | -11.29 to -2.425 | **** | <0.0001 |
| RLTR24\|Muridae | 4.643 | 0.2096 to 9.077 | * | 0.0221 |
| RLTR25A\|Muridae | -4.47 | -8.903 to -0.03595 | * | 0.0436 |
| RLTR26C_MM\|Mus_musculus | 7.233 | 2.799 to 11.67 | **** | <0.0001 |
| RLTR28\|Muridae | -5.98 | -10.41 to -1.547 | **** | <0.0001 |
| RLTR28B\|Muridae | -5.311 | -9.744 to -0.8773 | ** | 0.0013 |
| RLTR3_Mm\|Mus_mouse_genus | -7.418 | -11.85 to -2.984 | **** | <0.0001 |
| RLTR30\|Muridae | 10.39 | 5.955 to 14.82 | **** | <0.0001 |
| RLTR30D2_MM\|Mus_musculus | -5.293 | -9.726 to -0.8592 | ** | 0.0014 |
| RLTR31C_MM\|Mus_musculus | -6.41 | -10.84 to -1.976 | **** | <0.0001 |
| RLTR33\|Muridae | 5.447 | 1.014 to 9.881 | *** | 0.0007 |
| RLTR4_Mm\|Mus_mouse_genus | -14.01 | -18.44 to -9.576 | **** | <0.0001 |
| RLTR4_MM-int\|Mus_mouse_genus | -18.33 | -22.77 to -13.90 | **** | <0.0001 |
| RLTR44B\|Mus_mouse_genus | 9.766 | 5.332 to 14.20 | **** | <0.0001 |
| RLTR45\|Mus_mouse_genus | -8.113 | -12.55 to -3.679 | **** | <0.0001 |
| RLTR45-int\|Mus_mouse_genus | -8.27 | -12.70 to -3.836 | **** | <0.0001 |
| RLTR46\|Muridae | 9.782 | 5.349 to 14.22 | **** | <0.0001 |
| RLTR46B\|Mus_musculus | -4.984 | -9.417 to -0.5500 | ** | 0.0054 |
| RLTR49\|Mus_musculus | -4.612 | -9.045 to -0.1783 | * | 0.0251 |
| RLTR5_Mm\|Mus_mouse_genus | -5.973 | -10.41 to -1.540 | **** | <0.0001 |
| RLTR50B\|Mus_musculus | -7.971 | -12.40 to -3.537 | **** | <0.0001 |
| RLTR51A_Mm\|Mus_musculus | -5.406 | -9.839 to -0.9721 | *** | 0.0008 |
| RLTR6_Mm\|Mus_mouse_genus | -12.11 | -16.55 to -7.680 | **** | <0.0001 |
| RLTR6B_Mm\|Mus_musculus | -8.086 | -12.52 to -3.652 | **** | <0.0001 |
| RLTR6C_Mm\|Mus_musculus | -8.366 | -12.80 to -3.933 | **** | <0.0001 |
| RLTR6-int\|Mus_mouse_genus | -13.66 | -18.10 to -9.231 | **** | <0.0001 |
| RLTR8\|Mus_mouse_genus | 4.622 | 0.1884 to 9.056 | * | 0.0241 |
| RLTR9A3\|Mus_musculus | -6.859 | -11.29 to -2.426 | **** | <0.0001 |
| RLTR9C\|Mus_mouse_genus | 9.812 | 5.378 to 14.25 | **** | <0.0001 |
| RLTR9E\|Mus_mouse_genus | 8.744 | 4.310 to 13.18 | **** | <0.0001 |
| RLTR9F\|Mus_mouse_genus | -4.621 | -9.055 to -0.1877 | * | 0.0241 |
| RLTRETN_Mm\|Mus_mouse_genus | -10.47 | -14.90 to -6.036 | **** | <0.0001 |
| RMER10B\|Muridae | -7.315 | -11.75 to -2.882 | **** | <0.0001 |
| RMER12C\|Muridae | 5.41 | 0.9759 to 9.843 | *** | 0.0008 |
| RMER13B\|Muridae | -8.171 | -12.60 to -3.738 | **** | <0.0001 |
| RMER15-int\|Muridae | 5.315 | 0.8813 to 9.748 | ** | 0.0012 |
| RMER16B3\|Muridae | 8.175 | 3.741 to 12.61 | **** | <0.0001 |
| RMER16C\|Muridae | 10.55 | 6.116 to 14.98 | **** | <0.0001 |
| RMER16-int\|Muridae | 4.821 | 0.3871 to 9.254 | * | 0.0107 |
| RMER17A\|Mus_mouse_genus | -8.087 | -12.52 to -3.653 | **** | <0.0001 |
| RMER17A2\|Mus_mouse_genus | -8.664 | -13.10 to -4.231 | **** | <0.0001 |
| RMER17C\|Muridae | -5.584 | -10.02 to -1.150 | *** | 0.0004 |
| RMER17C2\|Muridae | -6.61 | -11.04 to -2.176 | **** | <0.0001 |
| RMER17D\|Muridae | -9.633 | -14.07 to -5.200 | **** | <0.0001 |
| RMER19B2\|Muridae | -6.202 | -10.64 to -1.768 | **** | <0.0001 |
| RMER1B\|Muridae | -5.477 | -9.910 to -1.043 | *** | 0.0006 |
| RMER1C\|Muridae | -5.701 | -10.13 to -1.268 | *** | 0.0002 |
| RMER21A\|Muridae | -7.97 | -12.40 to -3.537 | **** | <0.0001 |
| RMER3D1\|Muridae | 5.171 | 0.7373 to 9.605 | ** | 0.0024 |
| RMER3D2\|Muridae | 11.52 | 7.084 to 15.95 | **** | <0.0001 |
| RMER3D3\|Muridae | 15.71 | 11.27 to 20.14 | **** | <0.0001 |
| RMER3D4\|Muridae | 5.11 | 0.6767 to 9.544 | ** | 0.0031 |
| RMER3D-int\|Muridae | 8.367 | 3.934 to 12.80 | **** | <0.0001 |
| RMER6A\|Muridae | -6.182 | -10.62 to -1.748 | **** | <0.0001 |
| RMER6C\|Muridae | 6.15 | 1.717 to 10.58 | **** | <0.0001 |
| SRV_MM-int\|Mus_mouse_genus | -6.189 | -10.62 to -1.755 | **** | <0.0001 |
| SSU-rRNA_Hsa\|Metazoa | -5.233 | -9.666 to -0.7993 | ** | 0.0018 |
| Tigger12\|Mammalia | 7.05 | 2.617 to 11.48 | **** | <0.0001 |
| Tigger17a\|Mammalia | 5.418 | 0.9843 to 9.851 | *** | 0.0008 |
| Tigger17c\|Eutheria | 7.777 | 3.343 to 12.21 | **** | <0.0001 |
| Tigger5b\|Eutheria | 9.226 | 4.793 to 13.66 | **** | <0.0001 |
| Tigger8\|Eutheria | 7.374 | 2.940 to 11.81 | **** | <0.0001 |
| Tigger9a\|Mammalia | 5.884 | 1.451 to 10.32 | **** | <0.0001 |
| Tigger9b\|Eutheria | 14.11 | 9.672 to 18.54 | **** | <0.0001 |
| URR1B\|Muridae | 7.047 | 2.613 to 11.48 | **** | <0.0001 |
| Zaphod\|Eutheria | 7.726 | 3.292 to 12.16 | **** | <0.0001 |
| Zaphod3\|Eutheria | 9.741 | 5.307 to 14.17 | **** | <0.0001 |
| ZP3AR\|Muridae | -8.405 | -12.84 to -3.971 | **** | <0.0001 |

^†^p-values adjusted using Sidak’s multiple comparisons test
