# Supplementary material for: 5-Azacytidine Potentiates Anti-tumor Immunity in a Model of Pancreatic Ductal Adenocarcinoma
Source: Front Immunol. 2020 Mar 31;11:538. doi: 10.3389/fimmu.2020.00538 (PMC7136411; doi:10.3389/fimmu.2020.00538)
Supplement: Supplementary file 3 [file Table_3.docx]

**Supplemental Table 3:** Quantitative PCR Primer Sequences

**Mouse Targets**

| H2-D-F | 5’ – AGTGGTGCTGCAGAGCATTA – 3’ |  |
| --- | --- | --- |
| H2-D-R | 5’ – CCTTTGGGGAATCTGTGCG – 3’ |  |
| muB2m-F | 5’ – CCTGGTCTTTCTGGTGCTTG – 3’ |  |
| muB2m-R | 5’ – TTCAGTATGTTCGGCTTCCC – 3’ |  |
| IAP-F | 5’ – AAGCAGCAATCACCCACTTTGG – 3’ |  |
| IAP-R | 5’ – CAATCATTAGATGCGGCTGCCAAG - 3’ |  |
| muCXCL11- F | 5’ – GGCTTCCTTATGTTCAAACAGGG – 3’ |  |
| muCXCL11-R | 5’ – GCCGTTACTCGGGTAAATTACA – 3’ |  |

**Human Targets**

| HLA-A-F | 5’ – AAAAGGAGGGAGTTACACTCAGG – 3’ |  |
| --- | --- | --- |
| HLA-A-R | 5’ – AGCTGTCTCACACTTTACAAGC – 3’ |  |
| HLA-C-F | 5’ – CCTGGTTGTCCTAGCTGTCCTT – 3’ |  |
| HLA-C-R | 5’ – GCTCCCTCCTTTTCCACCTGA – 3’ |  |
| HuB2m-F | 5’ – TCCATCCGACATTGAAGTTG – 3’ |  |
| HuB2m-R | 5’ – ACACGGCAGGCATACTCAT - 3’ |  |
| HuTap1-F | 5’ – TCCTGGTGGTCCTCTCCTCTCTT – 3’ |  |
| HuTap1-R | 5’ – GTGTTGTTATAGATCCCGTCACCC – 3’ |  |
| HuLmp2-F | 5’ – GATGGGTTCTGATTCCCGAGTGT – 3’ |  |
| HuLmp2-R | 5’ – AGGTTCCTCCAGTTCTATCCC – 3’ |  |
| HuCXCL10-F | 5’ – AAATATGGCACACTAGCCCC – 3’ |  |
| HuCXCL10-R | 5’ – AGAGGTACTCCTTGAATGCC – 3’ |  |
