# Supplementary material for: 5-Azacytidine Potentiates Anti-tumor Immunity in a Model of Pancreatic Ductal Adenocarcinoma
Source: Front Immunol. 2020 Mar 31;11:538. doi: 10.3389/fimmu.2020.00538 (PMC7136411; doi:10.3389/fimmu.2020.00538)
Supplement: Supplementary file 4 [file Presentation_1.PPTX]

## Slide 1
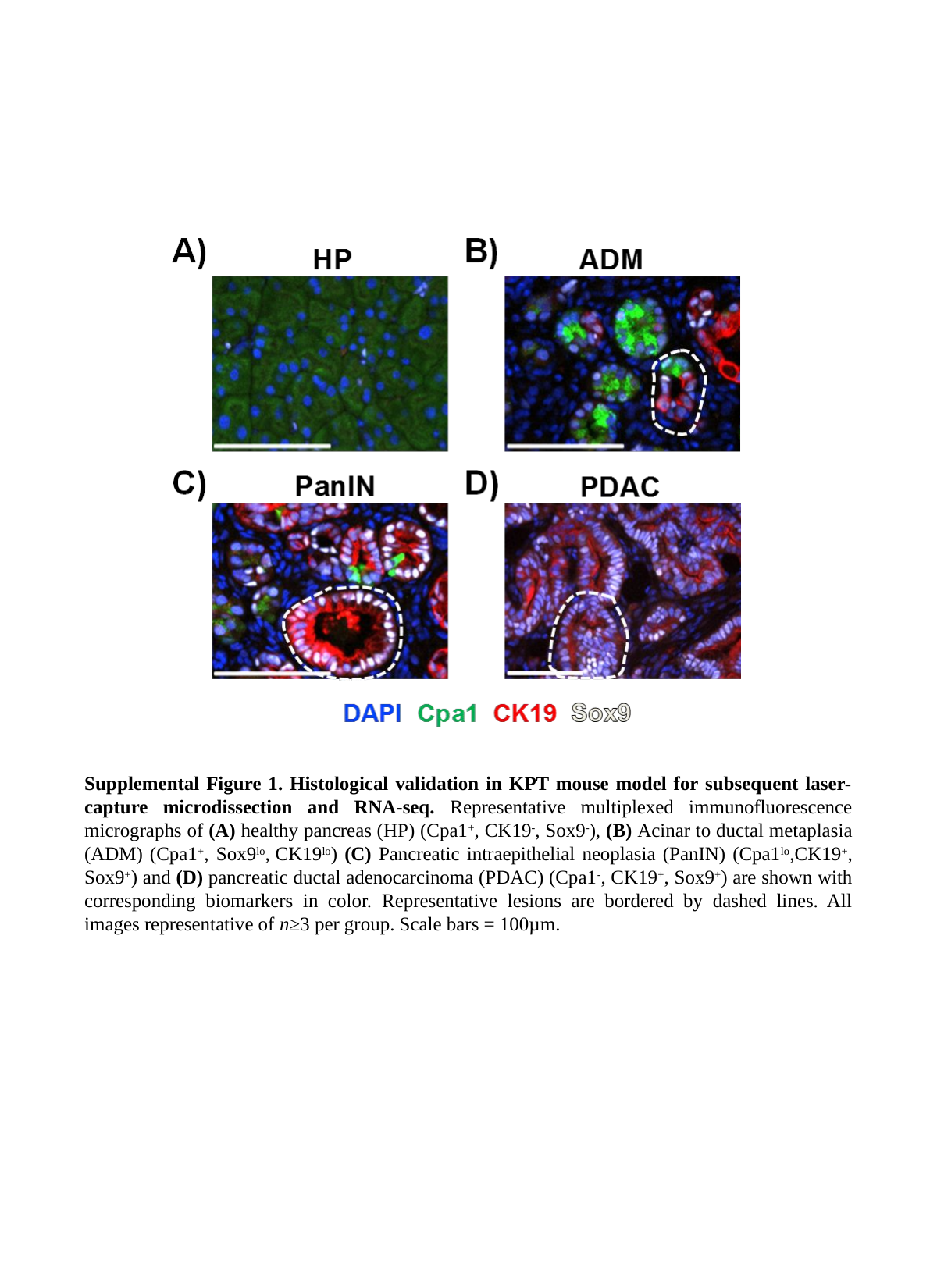

Supplemental Figure 1. Histological validation in KPT mouse model for subsequent laser-capture microdissection and RNA-seq. Representative multiplexed immunofluorescence micrographs of (A) healthy pancreas (HP) (Cpa1+, CK19-, Sox9-), (B) Acinar to ductal metaplasia (ADM) (Cpa1+, Sox9lo, CK19lo) (C) Pancreatic intraepithelial neoplasia (PanIN) (Cpa1lo,CK19+, Sox9+) and (D) pancreatic ductal adenocarcinoma (PDAC) (Cpa1-, CK19+, Sox9+) are shown with corresponding biomarkers in color. Representative lesions are bordered by dashed lines. All images representative of n≥3 per group. Scale bars = 100µm.

## Slide 2
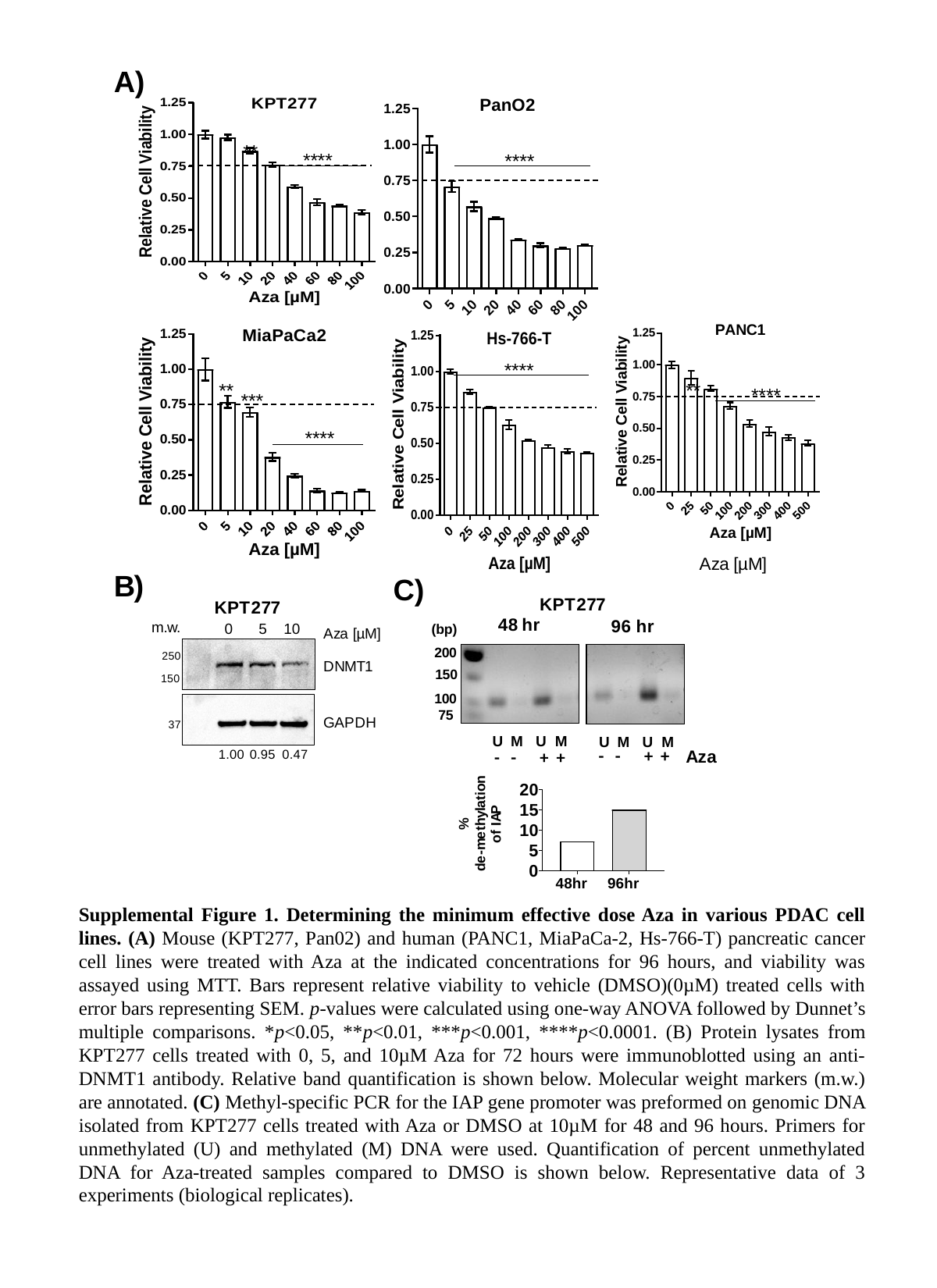

Supplemental Figure 1. Determining the minimum effective dose Aza in various PDAC cell lines. (A) Mouse (KPT277, Pan02) and human (PANC1, MiaPaCa-2, Hs-766-T) pancreatic cancer cell lines were treated with Aza at the indicated concentrations for 96 hours, and viability was assayed using MTT. Bars represent relative viability to vehicle (DMSO)(0µM) treated cells with error bars representing SEM. p-values were calculated using one-way ANOVA followed by Dunnet’s multiple comparisons. *p<0.05, **p<0.01, ***p<0.001, ****p<0.0001. (B) Protein lysates from KPT277 cells treated with 0, 5, and 10µM Aza for 72 hours were immunoblotted using an anti-DNMT1 antibody. Relative band quantification is shown below. Molecular weight markers (m.w.) are annotated. (C) Methyl-specific PCR for the IAP gene promoter was preformed on genomic DNA isolated from KPT277 cells treated with Aza or DMSO at 10µM for 48 and 96 hours. Primers for unmethylated (U) and methylated (M) DNA were used. Quantification of percent unmethylated DNA for Aza-treated samples compared to DMSO is shown below. Representative data of 3 experiments (biological replicates).

## Slide 3
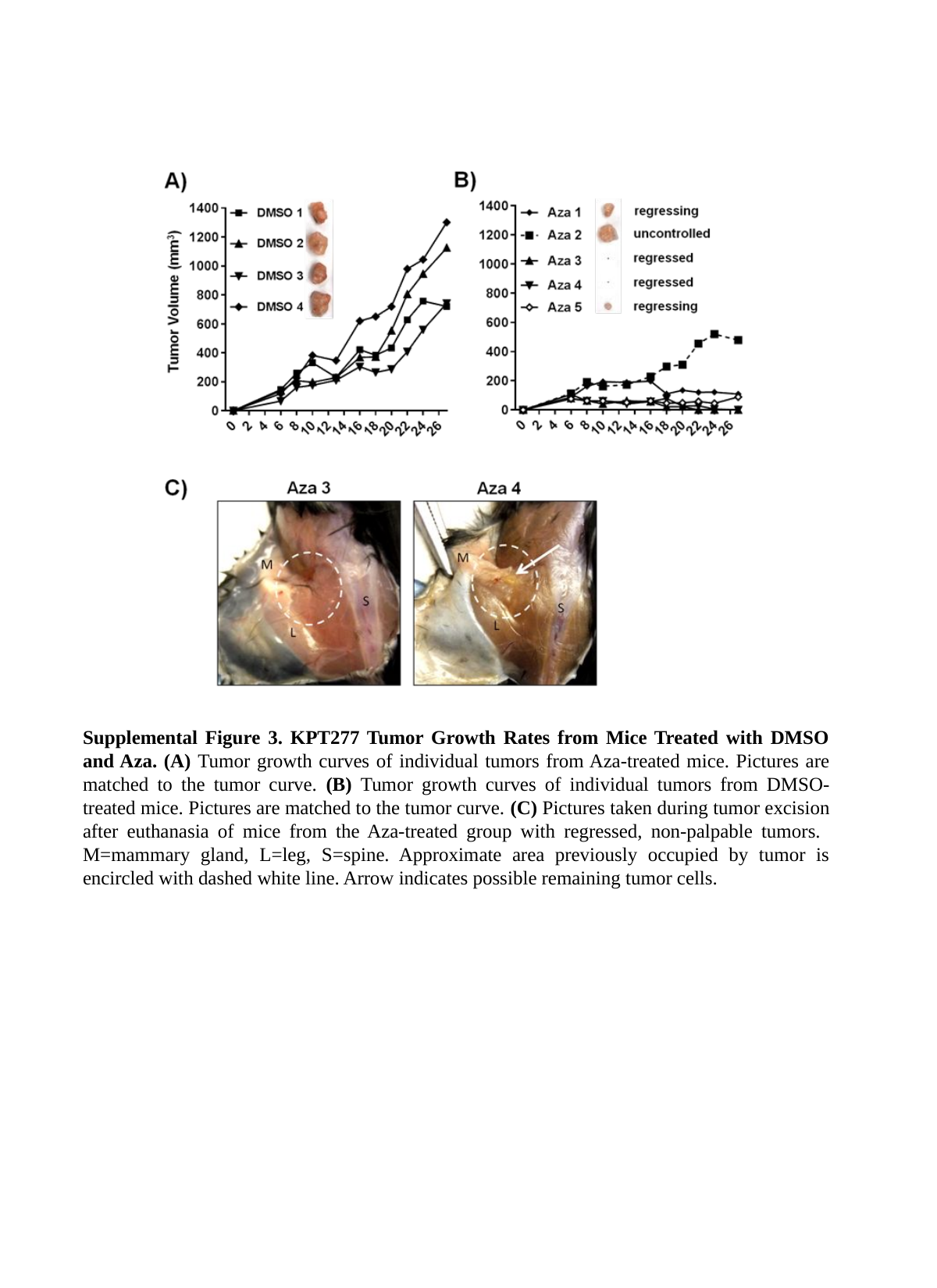

Supplemental Figure 3. KPT277 Tumor Growth Rates from Mice Treated with DMSO and Aza. (A) Tumor growth curves of individual tumors from Aza-treated mice. Pictures are matched to the tumor curve. (B) Tumor growth curves of individual tumors from DMSO-treated mice. Pictures are matched to the tumor curve. (C) Pictures taken during tumor excision after euthanasia of mice from the Aza-treated group with regressed, non-palpable tumors. M=mammary gland, L=leg, S=spine. Approximate area previously occupied by tumor is encircled with dashed white line. Arrow indicates possible remaining tumor cells.

## Slide 4
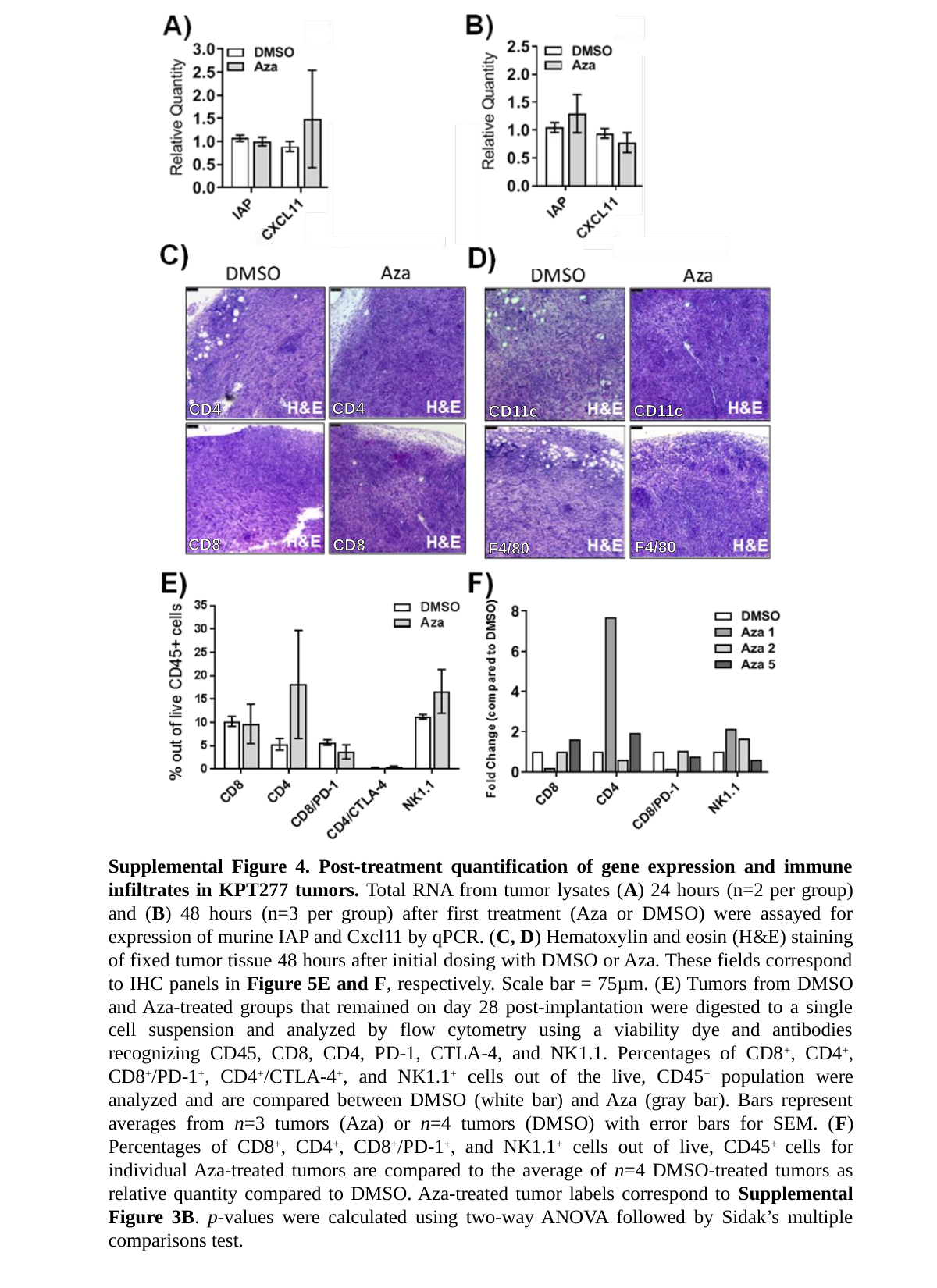

CD4
CD4
CD11c
CD11c
CD8
CD8
F4/80
F4/80
Supplemental Figure 4. Post-treatment quantification of gene expression and immune infiltrates in KPT277 tumors. Total RNA from tumor lysates (A) 24 hours (n=2 per group) and (B) 48 hours (n=3 per group) after first treatment (Aza or DMSO) were assayed for expression of murine IAP and Cxcl11 by qPCR. (C, D) Hematoxylin and eosin (H&E) staining of fixed tumor tissue 48 hours after initial dosing with DMSO or Aza. These fields correspond to IHC panels in Figure 5E and F, respectively. Scale bar = 75µm. (E) Tumors from DMSO and Aza-treated groups that remained on day 28 post-implantation were digested to a single cell suspension and analyzed by flow cytometry using a viability dye and antibodies recognizing CD45, CD8, CD4, PD-1, CTLA-4, and NK1.1. Percentages of CD8+, CD4+, CD8+/PD-1+, CD4+/CTLA-4+, and NK1.1+ cells out of the live, CD45+ population were analyzed and are compared between DMSO (white bar) and Aza (gray bar). Bars represent averages from n=3 tumors (Aza) or n=4 tumors (DMSO) with error bars for SEM. (F) Percentages of CD8+, CD4+, CD8+/PD-1+, and NK1.1+ cells out of live, CD45+ cells for individual Aza-treated tumors are compared to the average of n=4 DMSO-treated tumors as relative quantity compared to DMSO. Aza-treated tumor labels correspond to Supplemental Figure 3B. p-values were calculated using two-way ANOVA followed by Sidak’s multiple comparisons test.
